# Mechanotransduction in Shaping Immunity: Pathways, Crosstalk, and Pathophysiological Relevance

**DOI:** 10.1002/advs.202512164

**Published:** 2025-09-29

**Authors:** Ruijiao Yan, Yuyu Li, Shuo Chen, Li Zhu, Chenchen Zhou, Jianwei Chen, Shujuan Zou, Xianglong Han

**Affiliations:** ^1^ State Key Laboratory of Oral Diseases & National Center for Stomatology & National Clinical Research Center for Oral Diseases West China Hospital of Stomatology Sichuan University Chengdu 610041 China

**Keywords:** adaptive immune response, biomechanical cues, innate immune response, mechanical crosstalk, mechanotransduction

## Abstract

The immune system serves as a crucial line of defense against pathogens and abnormal cells throughout the entire life cycle. While chemical interactions among immune cells play a prominent role in the immune response, the manner in which physical factors, such as mechanical forces, shape the behaviors and functions of immune cells remains inadequately understood. This is particularly noteworthy given that the lifespan of an immune cell is characterized by a series of physical processes. This review first synthesizes current understanding of how immune cells respond to biomechanical cues via mechanotransduction cascades, which encompass mechanosensors that detect mechanical stimuli, mechanotransducers that propagate signals, and mechanoeffectors that execute cellular responses. Furthermore, this is delved into the mechanical crosstalk among immune cells themselves and between the extracellular matrix (ECM), as well as with other cell types. the emerging significance of mechanoimmunology is highlighted in both health and disease contexts. Overall, this review will give insight into the role of mechanotransduction in shaping immunity is hoped, providing inspiration for advancements in mechanobiology‐based immunotherapeutic strategies in the future.

## Graphical abstract

This review comprehensively summarizes the role of mechanotransduction in shaping immunity. It details how immune cells respond to biomechanical cues via mechanotransduction cascades and emphasizes the mechanical crosstalks within the immune microenvironment. The emerging significance of mechanoimmunology in both health and diseases is also highlighted. Collectively, these insights provide inspiration for the development of mechanobiology‐based immunotherapeutic strategies in the future.

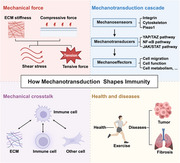

## 1 Introduction

The immune response serves as a fundamental defense mechanism that facilitates the maintenance of health in organisms by defending against pathogens, preserving homeostasis, and conducting immune surveillance.^[^ ^1^ ^]^ Dysregulation of the immune response, however, can result in diseases including cancer, fibrotic disorders, and autoimmune diseases.^[^ ^2^, ^3^ ^]^ As central orchestrators of the immune responses, immune cells mediate highly coordinated defense mechanisms through a sophisticated three‐tiered cascade composed of receptor recognition, signal transduction and effector function, ensuring antigen‐specific immunity while maintaining self‐tolerance.^[^ ^4^ ^]^ These cells possess the ability to sense and interpret signals from their microenvironment, enabling them to adapt their behaviors according to localized conditions. This ability is dominant throughout their lifespan, determining the fate of immune cells during developmental, homeostatic, and pathological processes.^[^ ^5^, ^6^ ^]^ Among the various factors that constitute immune microenvironment, chemical factors such as biochemical compositions (e.g., oxygen and ion concentrations) and soluble signaling molecules such as cytokines are well‐characterized, with their roles in modulating cellular behavior being extensively documented.^[^ ^7^, ^8^ ^]^ Although the immune response is primarily driven by chemical interactions among cells or between cells and pathogens, it is important to recognize that the life of an immune cell involves a dynamic orchestration of physical processes. These physical processes encompass extensive morphological changes, rapid migration through tight interstitial spaces, shear‐resistant adhesion to vascular endothelium, and the formation of specialized intercellular junctions with other cells.^[^ ^9^ ^]^ As a subset of physical factors, mechanical forces are emerging to be critical modulators of diverse physiological and pathological processes.^[^ ^10^, ^11^, ^12^ ^]^ However, mechanical forces within the immune microenvironment have received comparatively less attention.

In immune microenvironment, mechanical forces including tension, compression, shear stress, and hydrostatic pressure, regulate activities of immune cells through mechanisms that depend on the physical properties of neighboring cells, the mechanics of the extracellular matrix (ECM), and other microenvironmental factors.^[^ ^13^, ^14^, ^15^ ^]^ For instance, the ECM stiffness could modulate macrophages polarization through the NF‐κB signaling pathway mediated by the reactive oxygen species (ROS).^[^ ^16^ ^]^ Specifically, the stiffness of collagen fibers promoted M1 macrophage polarization (inflammatory phenotype), while the stiffness of osteoid contributed to M2 macrophage polarization (anti‐inflammatory phenotype). Immune cells that are mechanosensitive can transduce mechanical stimuli into biochemical or electrical signals through a biological process called mechanotransduction. Mechanotransduction is initiated by specialized force‐sensing proteins that serve as mechanosensors. Key examples include integrins, which bind to the ECM and sense its stiffness and topography, as well as Piezo1 channel and transient receptor potential vanilloid 4 (TRPV4) channel, which respond to membrane tension and shear stress.^[^ ^17^, ^18^ ^]^ Upon force application, these mechanosensors undergo conformational changes or clustering. This mechanical perturbation triggers a cascade of biochemical signaling events. For instance, force‐induced integrin activation recruits adaptor proteins (e.g., talin, vinculin) and activates focal adhesion kinase (FAK) and Src family kinases, leading to downstream activation of Rho GTPases (RhoA, Rac1, Cdc42) and reorganization of the actin cytoskeleton.^[^ ^19^, ^20^ ^]^ Similarly, Piezo1 channel opening allows rapid Ca^2^⁺ influx, a potent second messenger.^[^ ^21^ ^]^ These force‐triggered signaling pathways enable immune cells to adapt their behavior, structure, or function accordingly.^[^ ^20^, ^22^ ^]^ For better understanding, we summarize a network of mechanotransduction consisting of mechanosensors, mechanotranducers and mechanoeffectors. First, mechanosensitive immune cells sense mechanical stimuli through mechanosensors such as ion channel, integrin and cytoskeletons. Then, mechanotransducers undergo conformational changes and dynamical relocalization in respond to upstream signals from sensors.^[^ ^18^, ^23^, ^24^ ^]^ Ultimately, mechanoeffectors respond to these series of mechanical cues in cellular behavior, tissue homeostasis, and pathophysiological outcomes. Such mechanotransduction cascades enable immune cells to orchestrate spatiotemporally responses to external mechanical forces.

Herein, we systematically reviewed the role of mechanotransduction in shaping immune cells. First, we discussed the mechanotransduction cascades in immune cells, focusing on the underlying mechanisms of how mechanical forces affect immune cells (innate immune cell and adaptive immune cells). Then, we highlighted the mechanical crosstalk in immunity from three aspects: the crosstalk between immune cells and ECM, the crosstalk between immune cells, as well as the crosstalk between immune cells and other cells. Furthermore, the role of mechanoimmunology in health and diseases was mentioned. Our aim was to provide novel perspectives and conceptual frameworks for the discovery of new avenues at the intersection of cellular mechanics, mechanotransduction, and immunity, thereby highlighting the emerging directions for interdisciplinary research and therapeutic innovation for mechanoimmunology‐related diseases.

## 2 Mechanotransduction in immune cells

### 2.1 Mechanotransduction cascades in immune cells

**Figure 1:**
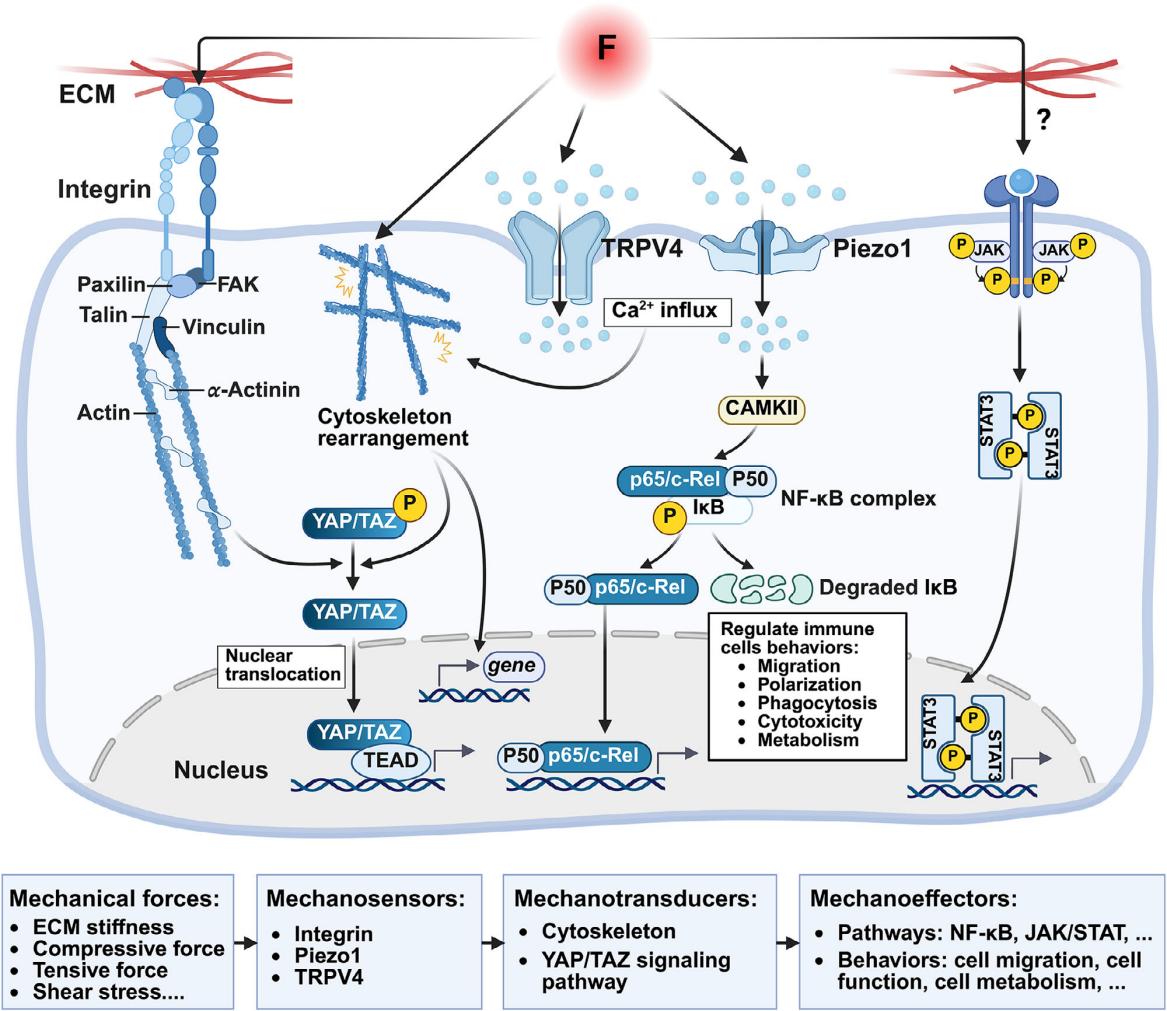
Schematic illustration of mechanotransduction cascades in immune cells. Mechanical forces (ECM stiffness, compressive force, tensive force and shear stress) act on immune cells, triggering mechanotransduction cascades. First, mechanosensors including integrins, Piezo1 and TRPV4 detect mechanical stimuli, converting mechanical signals into chemical or electrical signals via ion influx and conformational changes. Subsequently, downstream mechanotransducers such as cytoskeletons and YAP/TAZ, are activated in the cytoplasm. Finally, mechanoeffectors serve as the ultimate output of mechanical stimuli, including the activation of NF‐κB and STAT signaling, as well as the changes of immune cell behaviors such as migration, functional activity, and metabolism. This figure was created with BioRender.com.

In this part, we summarized mechanotransduction of immune cells with three‐tiered cascades including mechanosensors, mechanotranducers and mechanoeffectors (**Figure** **1**). Specifically, mechanosensors are defined as sensory elements within the cell membrane or cytoplasm which are sensitive to mechanical stimuli and convert mechanical signals into chemical or electrical signals, including integrins, cytoskeletons and Piezo1. Mechanotransducers respond to upstream signals from sensors and regulate gene expression of immune cells. Mechanoeffectors, generated as the end‐results of mechanical stimulation, induce changes in cellular behaviors such as migration, polarization, phagocytic activity, cytotoxicity, and metabolic adjustments. The three‐tiered cascades of mechanotransduction in immunity will be elaborated below.

#### 2.1.1 Mechanosensors

##### Integrins

Integrins constitute a superfamily of heterodimeric transmembrane receptors, composed of non‐covalently associated α and β subunits. They play critical roles in mediating cell‐ECM interactions, and facilitating cell‐cell communications.^[^ ^25^, ^26^ ^]^ It is reported that leukocytes adhered to vascular endothelium and migrated to the inflammatory site with the help of integrins. Tumor cells altered the expression of integrins to facilitate their invasion into surrounding tissues.^[^ ^27^, ^28^ ^]^ Besides, integrins were found to act as mechanical sensors by converting mechanical cues of ECM (rigidity, tension, topography) into biochemical signals through conformational activation and clustering.^[^ ^29^, ^30^ ^]^ For example, integrins could be activated under stiff ECM which contributed to enhanced phagocytosis of macrophages.^[^ ^31^ ^]^ Such mechanosensitive ability enables integrins to regulate the mechanical response of immune cells, dynamically coordinating their activation, migration, polarization and effector functions.

Emerging evidence has highlighted integrins as critical mechanosensors in enabling T cells to sense and respond to mechanical stimuli. Lymphocyte function‐associated antigen‐1 (LFA‐1) is a typical integrin expressed on the surface of T cells, which stands out for being especially attuned to detecting and delivering forces. LFA‐1 contributed to form synaptic subdomains with intercellular adhesion molecule‐1 (ICAM‐1) on the target cell surface, thus promoting the targeted release of cytotoxic particles including perforin and granzyme.^[^ ^32^ ^]^ Under tensive force, mechanically active LFA‐1 bound to ICAM‐1 more easily, leading to the conformational changes of downstream talin and rearrangements of cytoskeleton. As a result, lytic granules, a kind of specialized secretory lysosomes storing perforin and granzyme, were induced to fuse and the cytotoxicity of T cells was facilitated.^[^ ^33^ ^]^ Another study further elucidated the mechanotransductive interplay between LFA‐1/ICAM‐1 complex and T cell receptor (TCR) in regulating T cell activation thresholds.^[^ ^34^ ^]^ The results showed that the mechanical force transduction through the LFA‐1/ICAM‐1 complex exceeding a threshold of 12 pN potentiated the activation of antigen‐dependent T cells and enhanced TCR discrimination when stimulated by near cognate antigens. Integrins was also reported to engage in affecting the behaviors of macrophages. Hu et al. examined how integrins regulated the phagocytic receptor FcγR‐mediated phagocytosis of macrophages and revealed that, upon FcγR activation, the integrins formed a “mechanical barrier” around the macrophages, physically restricting the phosphatase CD45 from the phagocytic cup, a process critical for optimizing phagocytosis.^[^ ^31^ ^]^ Besides, substrate stiffness and roughness, which mimicked the ECM microenvironment in vitro, could also promote the phagocytosis of macrophages by the formation of integrin‐regulated focal adhesions and cytoskeletal reorganization.^[^ ^35^ ^]^

##### Piezo1

Exposure of immune cells to mechanical stimuli triggers ions influx through channels on the plasma membrane.^[^ ^36^ ^]^ Piezo1, a mechanosensitive ion channel that is distributed widely within the body, has emerged as a critical mediator in the mechanotransduction process of key immune populations including dendritic cells (DCs), macrophages, and T cells.^[^ ^37^ ^]^ Once detecting mechanical cues, Piezo1 becomes activated and opens, allowing the influx of a substantial number of extracellular cations, with Ca^2+^ being representative. This mechanotransduction event subsequently triggers a cascade of downstream reactions that modulate immune responses.^[^ ^38^ ^]^

Changes of ECM stiffness are the key mechanical features of dynamic cell‐microenvironment interactions that profoundly affect immune cell behaviors.^[^ ^39^, ^40^ ^]^ Basically, Piezo1 could directly sense membrane tension from ECM via mechanical deformation of their transmembrane helices. The mechanical forces transduced through lipid bilayers to the channel core of Piezo1, inducing conformational rearrangements for Piezo1 opening.^[^ ^41^, ^42^ ^]^ Moreover, ECM could directly regulate the conformation of integrins thus promoting actin polymerization and stress fiber formation via FAK activation. The contractile force of stress fibers is transmitted to the cell membrane via focal adhesions, further increasing local tension and synergistically activating Piezo1.^[^ ^43^, ^44^ ^]^ It has been demonstrated that immune cells are capable to sense ECM stiffness through Piezo1. For instance, Piezo1 could regulate the polarization of macrophages by sensing microenvironmental stiffness.^[^ ^45^ ^]^ Under stiff substrates, Piezo1 activity was enhanced in macrophages with large Ca^2+^ influx ([Ca^2+^]_i_). As a result, the activation of Piezo1 in macrophages enhanced macrophage interferon‐γ (IFN‐γ)‐ and lipopolysaccharide (LPS)‐induced inflammation as well as delayed the wound healing response. Likewise, the expression of Piezo1 was elevated in hepatic fibrosis, a typical type of pathological ECM stiffness.^[^ ^46^ ^]^ Compared to soft substrates, macrophages showed enhanced efferocytosis via the activation of Piezo1 when cultured on rigid substrates. This suggested that Piezo1 activation was necessary for stiffness‐dependent efferocytosis in macrophages. In DCs, Piezo1 was activated under stiff substrate thus engaging in the regulation of glucose metabolism of DCs.^[^ ^47^ ^]^ While direct comparative studies on how the dimensionality of ECM stiffness affects Piezo1 activation in immune cells remain limited, it is plausible to hypothesize that cells embedded within a 3D ECM microenvironment, which allows greater spatial interaction with the matrix, may exhibit enhanced mechanical coupling and thus more Piezo1 activation in response to ECM stiffness, compared to cells cultured on 2D substrates.

**Figure 2:**
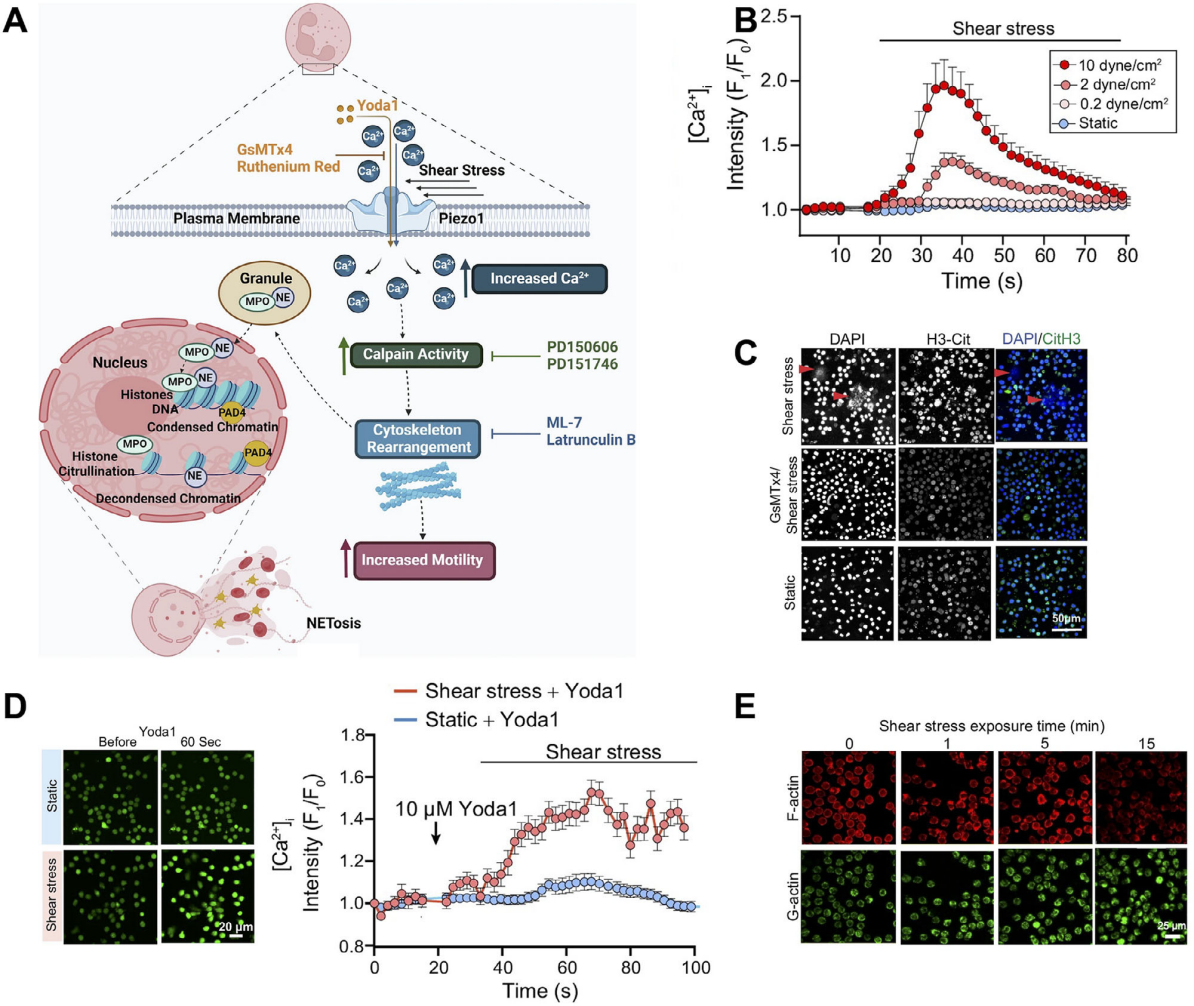
Piezo1 acted as a mechanosensor in neutrophils. A) Illustration of Piezo1‐mediated NETosis of neutrophils under shear stimulation. Upon the stimulation of shear stress, Piezo1‐mediated Ca^2+^ influx triggered the activation of calpain, thereby inducing cytoskeletal rearrangement and promoting enhanced motility and NETosis in neutrophils. B) Higher shear stress induced a larger [Ca^2+^]_i_ in neutrophils. Representative time‐lapse of [Ca^2+^]_i_ fluorescence in neutrophils under shear stress. C) Shear stress could induce the NET release (red arrows), which could be inhibited by the Piezo1 inhibitor, GsMTX4. Representative fluorescent images depicting the degree of NETosis and H3 citrullination (H3‐Cit) in neutrophils under static conditions, shear stress stimulation, or Piezo1 inhibition (GsMTX4). D) The activation of Piezo1 required the stimulation of shear stress. Representative fluorescent images and time‐course data of [Ca^2+^]_i_ fluorescent intensity in response to Yoda1. E) Shear stress had effects on cytoskeleton remodeling in neutrophils. Representative confocal images of neutrophils subjected to shear stress for varying durations, stained with Alexa Fluor 488 DNase I (G‐actin, green) and Alexa Fluor 568 phalloidin (F‐actin, red). Reproduced under terms of the CC‐BY‐NC‐ND license.^[^ ^48^ ^]^ Copyright 2024, The Authors, published by Nature Publishing Group UK.

Besides ECM stiffness, other mechanical cues can also activate Piezo1 in immune cells. For example, shear stress could directly activate Piezo1 by elevating the local tension of cell membrane which ultimately regulated the behaviors of immune cells. During blood circulation, neutrophils undergo continuous hemodynamic forces particularly shear stress. Baratchi et al. demonstrated that shear stress triggered neutrophil extracellular trap formation (NETosis) through Piezo1‐dependent pathway (**Figure** **2**).^[^ ^48^ ^]^ Specifically, after shear stress stimulation, Piezo1‐mediated Ca^2+^ influx triggered calpain activation, thereby inducing cytoskeletal rearrangement and promoting enhanced motility and NETosis in neutrophils. Functioning as critical sentinels of the innate immune system, inflammasomes are responsible for sensing intracellular danger signals, participating in the activation of inflammatory responses. Studies revealed that the NOD‐like receptor family, pyrin domain containing 3 inflammasome could be enhanced via upregulating Piezo1 in macrophages when exposed to shear stress.^[^ ^49^ ^]^ In addition, it was uncovered that Piezo1 fully engaged in the migration of polymorphonuclear leukocytes (PMNs) by acting as a vital mechanosensor.^[^ ^50^ ^]^ During trans‐endothelial migration, PMNs sensed tensive forces by Piezo1 and induced the expression of Nox4, a kind of NADPH oxidase isoforms which was helpful to generate ROS. Consequently, transmigrating PMNs performed an enhanced bactericidal function through the activation of Piezo1‐Nox4 signaling pathway. During the adaptive immune responses, Piezo1 selectively suppressed the expansion of regulatory T cells without affecting the functions of CD4^+^ T cells.^[^ ^51^ ^]^ However, Yang et al. found that Piezo1 was critical for regulating the differentiation of interleukin‐9‐producing CD4^+^ T cells (Th9).^[^ ^52^ ^]^ This inconsistency implied that the involvement of Piezo1 in the mechanotransduction process of CD4^+^ T cells remained unclear. As an important role in antitumor immunity, cytotoxic T cells were able to increase their traction force and enhance their cytotoxicity when they encounter stiffened cancer cells.^[^ ^53^ ^]^ Pang et al. reported that the blockade of Piezo1 strengthened the traction forces of cytotoxic T cells and augmented their cytotoxicity against tumor cells, indicating that Piezo1 could be a potential mechanical regulator in antitumor therapy.^[^ ^54^ ^]^

##### TRPV4

Transient receptor potential vanilloid 4 (TRPV4) is a non‐selective cation channel permeable to Ca^2+^, Na^+^, and Mg^2+^ ions. TRPV4 is widely distributed in the cardiovascular system, kidney, skeletal system, inner ear, and other tissues.^[^ ^20^, ^55^ ^]^ It can sense various extracellular physical stimuli, including osmotic pressure changes, temperature changes, and mechanical force stimuli.^[^ ^56^, ^57^ ^]^ Similar to Piezo1, TRPV4 sensed the mechanical stimuli through the deformation of surrounding membrane (local tension force) which led to the channel opening. Dutta et al. clarified that TRPV4 engaged in the matrix stiffness‐induced macrophages polarization.^[^ ^58^ ^]^ When macrophages were embedded in stiff matrix of 50 kPa, the markers of M1 subtype *Il1β* and *Mcp1* were elevated in a TRPV4‐dependent manner. Du et al. carried research on the role of tissue resident (CCR2^−^) macrophages in dilated cardiomyopathy harboring.^[^ ^59^ ^]^ They found that TRPV4 sensed the mechanical stretch and engaged in the regulation of pro‐angiogenic growth‐factor expression in CCR2^−^ macrophages, which therefore explained the importance of CCR2^−^ macrophages in the maintenance of adaptive ventricular remodeling and the promotion of coronary angiogenesis. All this suggested that TRPV4 was a prominent mechanosensor in immune cells.

#### 2.1.2 Mechanotransducers

##### Cytoskeleton

The cytoskeleton is a dynamic network of protein filaments in eukaryotic cells, composed of microtubules, microfilaments and intermediate fibers.^[^ ^60^, ^61^ ^]^ It allowed immune cells to migrate and remodel their shapes via dynamic reorganization of actin networks, particularly during processes such as the generation of neutrophil extracellular traps (NET) and the phagocytic activity of macrophages.^[^ ^62^, ^63^ ^]^ Recent studies have revealed that the cytoskeleton not only provides structural scaffolding for cellular architecture but also acts as a mechanotransduction apparatus with intrinsic mechanosensitive properties, capable of transmitting, amplifying, and integrating mechanical signals initiated by mechanosensors. It was reported that the stiffness of ECM could promote the reorganization of actin.^[^ ^64^ ^]^ During toll‐like receptors (TLRs) signaling‐mediated innate immune responses of macrophages, LPS stimulation induced the assembly of Piezo1 on macrophages and TLR4 on pathogens, triggering cytoskeletal remodeling that enhanced phagocytic capacity and promoted bacterial clearance.^[^ ^65^ ^]^ Besides, the cytoskeletons exert function in both activation and immune synapse formation of B cells. It showed that the actin cytoskeleton was closely related to the organization of B cell receptor (BCR) oligomers into signaling‐competent microdomains, and the lack of the actin cytoskeleton might affect B cell activation.^[^ ^66^ ^]^ In immune synapse (IS) of B cells, the coordination of peripheral forces exerted by myosin II as well as central forces exerted by actin drove antigen extraction of B cells, uncovering an actomyosin‐driven force patterning in B cells.^[^ ^67^ ^]^ While the actin cytoskeletons were the main participants in B cells activation IS formation, whether they participated in the integrin‐dependent IS formation was unknown. Wang et al. demonstrated that during integrin‐dependent IS formation, inhibition of myosin compromised synapse formation by reducing antigen centralization, diminishing BCR signaling, and disrupting the distribution of signaling proteins at the synapse.^[^ ^68^ ^]^ This suggested that the cytoskeletons assisted integrins in integrin‐dependent IS formation. Interestingly, it was discovered that cytoskeletons could serve as a potential mechanical sensor in macrophages as well. Meizlish et al. uncovered that the cytoskeleton could mediate macrophage mechanosensing via a noncanonical, integrin‐independent mechanism, which ultimately modify chromatin availability to regulate mechanosensitive gene expression.^[^ ^69^ ^]^

##### YAP/TAZ signaling pathway

Yes‐associated protein (YAP) and transcriptional coactivator with PDZ‐binding motif (TAZ), the terminal effectors of the Hippo signaling cascade, function as key transcriptional regulators that orchestrate cell proliferation, differentiation, and tissue homeostasis.^[^ ^70^ ^]^ Upon activation through dephosphorylation, YAP/TAZ translocate from the cytoplasm to the nucleus from where they bind transcription factors like the TEA domain transcription factor (TEAD) family, thereby directly regulating target gene expression.^[^ ^71^ ^]^ While the activity of YAP/TAZ is mainly regulated by Hippo‐dependent pathway, recent studies have demonstrated that YAP/TAZ could be directly influenced by mechanical stimuli. It was documented that YAP/TAZ participated in the mechanotransduction process especially in cancer and their aberrant activation was a hallmark of several malignancies such as hepatocellular carcinoma and breast cancer.^[^ ^72^, ^73^ ^]^ Aberrant mechanical signals in cancer, such as increased compressive forces, altered ECM composition, and elevated ECM rigidity, could stimulate YAP/TAZ activity. Notably, when cells were cultured on a stiff ECM, YAP/TAZ translocated to the nucleus and exhibited transcriptional activity; in contrast, on a soft ECM, these proteins were inhibited and localized to the cytoplasm. The activation of YAP/TAZ not only can be influenced by mechanical stimuli but also are critical regulators of immune responses, thereby linking mechanical cues to immunological functions.^[^ ^74^, ^75^, ^76^ ^]^ For example, highly fibrotic tissue has the capability to trigger persistent activation of YAP/TAZ in macrophages, and in turn the activation of YAP/TAZ leads to tissue repair or the aggravation of inflammation. According to a recent study, the fibrotic activities induced by M2 macrophages were attenuated through the inhibition of YAP/TAZ. This finding underscored the critical role that YAP/TAZ played in the fibrosis process mediated by macrophages.^[^ ^77^ ^]^ In another study, Mia et al. found that YAP/TAZ might be an important regulator of macrophages in post‐myocardial infarction (MI).^[^ ^78^ ^]^ As the results showed, when YAP/TAZ was deleted in macrophages, the pro‐inflammatory response was inhibited and the reparative response was enhanced. These data suggested that YAP/TAZ could serve as mechanical switches orchestrating macrophage phenotype and plasticity in different tissue contexts. In T cells, the stiffness of ECM modulates YAP/TAZ activity, which in turn influences T cell activation, proliferation, and function. It has been reported that lymph nodes transiently stiffened during immune activation, and YAP functioned to modulate NFAT1 nuclear translocation, metabolism, and effector differentiation of T cells in a stiffness‐dependent manner.^[^ ^79^ ^]^ In addition, regulatory T cells (Treg cells) function as critical immunomodulatory mediators to suppress excessive immune response and restrict pathological tissue remodeling after myocardial injury. Ramjee et al. found that YAP/TAZ‐deficient mice exhibited reduced Treg cells accumulation in the injured myocardium, causing severe post‐MI myocardial fibrosis.^[^ ^80^ ^]^ This implied that YAP/TAZ signals might contribute to adaptive immune regulation during the post‐MI recovery phase. Moreover, YAP/TAZ were capable to respond to upstream signaling from Piezo1 and transduce mechanical messages to downstream by regulating gene expression.^[^ ^81^ ^]^ Research suggested that following the activation of Piezo1 under stiff ECM stimulation, YAP/TAZ was dephosphorylated and translocated into nucleus and bound to TEAD, leading to an enhancement of glucose metabolism in DCs, which is pivotal to the development and function of DCs. And TAZ pharmacological or genetic inhibition reduced stiffness‐mediated inflammatory cytokine production by DCs.^[^ ^47^, ^82^, ^83^ ^]^ Mechanistically, YAP/TAZ integrate diverse forms of mechanical stimulation including ECM stiffness and regulate transcriptional programs controlling cytokine/chemokine production, and metabolic enzymes. These transcriptional outputs govern immune cell fate and function. In turn, immune cells remodel their surrounding matrix and mechanical landscape through secreting matrix metalloproteinases, aligning collagen fibers, and other mechanisms, which may contribute to the interaction of YAP/TAZ‐mediated mechanotransduction and immunity.^[^ ^84^, ^85^, ^86^ ^]^ Moreover, YAP/TAZ signaling intersects with canonical immune pathways such as tansforming growth factor‐β (TGF‐β), Wnt, MAPK, and PD1/PDL1 signaling, together fine‐tuning the balance between inflammation, tissue repair, and immune evasion.^[^ ^87^ ^]^

### 2.2 How biomechanics modulates immune cells

#### 2.2.1 Innate immune cells

**Figure 3:**
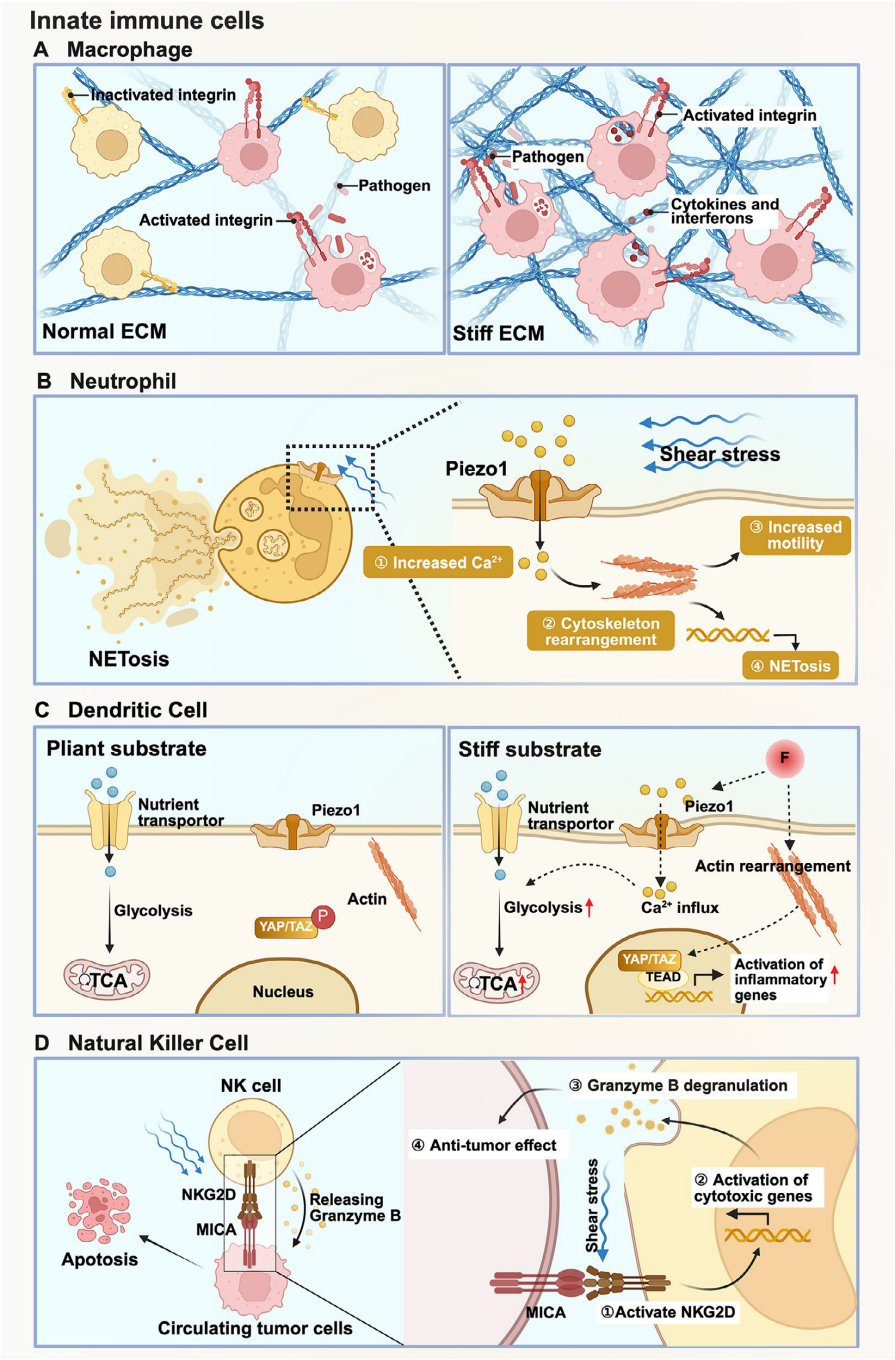
Examples of mechanical signaling regulating innate immune cells fate. A) Stiffer ECM enhanced the phagocytosis of macrophages through activating integrins. B) Shear stress increased the mobility of neutrophils and triggered NETosis in neutrophils. Under the stimulation of shear stress, mechanosensitive Piezo1 opened with an increase of Ca^2+^ influx. In response, the cytoskeletons rearranged which consequently contributed to enhance NETosis as well as the mobility of neutrophils. C) Stiff substrate heightened the glucose metabolism as well as pro‐inflammatory activities of DCs. In comparison to pliant substrate, stiff substrate activated Piezo1 of DCs, followed by the Ca^2+^ influx and upregulation of glycolysis. Moreover, the actin cytoskeletons rearranged in response to stiff substrate, thereby promoting the nuclear translocation of YAP/TAZ and initiating the inflammatory response in DCs. D) Fluid shear stress induced the activation and degranulation of NK cells, enhancing their anti‐tumor effect. In NK cells, the mechanosensitive receptor NKG2D first recognized MICA on tumor cells and was activated under shear stress. This mechanical signal was then transduced to modulate the expression of cytotoxic genes in NK cells, thereby enhancing granzyme B release of NK cells against tumor cells. This figure was created with BioRender.com.

As the body's frontline defense, innate immunity forms an evolutionarily conserved barrier that immediately constrains pathogen invasion, employing mechanisms such as phagocytic clearance through macrophages and neutrophils, cytotoxic elimination via natural killer (NK) cells, and antigen presentation by DCs.^[^ ^88^ ^]^ Recent studies have shown that in addition to being regulated by biochemical factors, innate immune cells exhibit particular sensitivity to mechanical stimuli.^[^ ^89^ ^]^ This underscores the evolving paradigm of innate immune cells as dynamic sensors capable of integrating biomechanical signals from their microenvironment to orchestrate context‐specific immune responses (**Figure** **3**).

As the most mechanically responsive innate immune cells, macrophages are capable of sensing and responding to biomechanical cues within their microenvironment. The phagocytic function of macrophages is directly regulated by ECM stiffness. For instance, when incubated in the stiff substrate, macrophages performed an enhanced phagocytosis against pathogens through integrin‐mediated mechanotransduction pathway.^[^ ^31^, ^35^ ^]^ In atherosclerosis, Piezo1 activation could promote the phagocytic activity of macrophages.^[^ ^90^ ^]^ The functional plasticity of macrophages allows them to adopt pro‐inflammatory (M1) or anti‐inflammatory (M2) phenotypes, namely the polarization of macrophages. According to phenotype and function, the polarization of macrophages can be classified into classically activated M1 macrophages and alternatively activated M2 macrophages, where the latter can be further divided into M2a, M2b, M2c and M2d subtypes.^[^ ^91^ ^]^ The polarization of macrophages is now recognized to be heavily influenced by mechanical cues. Stiff microenvironments, such as those in fibrotic lungs, promoted M2 polarization, which could be reversed by the inhibition of mechanosensory integrin.^[^ ^92^ ^]^ Serving as a key mechanoeffector in macrophages, the NF‐κB pathway are responsive to ECM mechanics, driving inflammatory responses.^[^ ^93^ ^]^ Chen et al. reported that low‐stiffness ECM could promote M1 polarization through enhanced phosphorylation of NF‐κB components.^[^ ^16^ ^]^ Similarly, the tumor‐associated macrophages were repolarized from M2 to M1 polarization under the elasticity of soft nanoparticles, initiating inflammatory responses through Piezo1‐related NF‐κB pathway.^[^ ^94^ ^]^ Mechanically, the soft elastic nanoparticles activated mechanosensitive Piezo1 on tumor‐associated macrophages, resulting in a large amount of Ca^2+^ influx, which followed by the activation of calcium/calmodulin‐dependent kinase II and the promotion of phosphorylated p65 in NF‐κB pathway. These findings highlight macrophages as integral mechanosensors that translate physical cues into immunomodulatory outcomes.

Neutrophils, another critical phagocytic innate immune cells, also exhibit mechanosensitivity, particularly in migration, cytokine secretion, and NETosis.^[^ ^95^ ^]^ They were able to employ migration strategies and dynamic trail‐formation mechanisms via cytoskeletal reorganization under varying adhesive conditions, which illustrated their mechanical adaptability.^[^ ^96^ ^]^ Nevertheless, the inflammatory regulation of neutrophils under stiff ECM is contradictory. Jiang et al. designed a 3D matrix composed of hydrogels to mimic different ECM stiffness around neutrophils.^[^ ^97^ ^]^ The findings showed that stiffer 3D matrix promoted neutrophil polarization toward an anti‐inflammatory phenotype through JAK1/STAT3 pathway. In mechanism, after JAK was activated by stiff substrates, the STAT was phosphorylated and translocated from cytoplasm into nucleus, which promoted the anti‐inflammatory genes expression in neutrophils. In contrast, Abaricia et al. found that neutrophils on 2D substrates with higher stiffness increased NETosis, as well as elevated secretion of pro‐inflammatory cytokines and chemokines.^[^ ^98^ ^]^ These divergent responses highlighted how neutrophil behavior was critically influenced by whether the mechanical environment was 2D or 3D.

DCs and NK cells also respond to mechanical stimuli, though research in these areas is still emerging. Generally, DCs are divided into immature DCs and mature DCs. The former function in antigen uptake and recognition, and the latter serve as professional antigen‐presenting cells (APCs), which process foreign antigens and present them to T cells, initiating adaptive immune responses.^[^ ^99^ ^]^ The dimensionality of ECM stiffness could affect DCs’ behaviors. When incubated on 2D stiff substrates, immature DCs performed enhanced proliferation, activation, and cytokine production through Hippo signaling pathway. In contrast, within a 3D stiff ECM environment, immature DCs display reduced motility, navigating more slowly through the matrix.^[^ ^100^ ^]^ Piezo1 might be the potential mechanosensor of immature DCs. The mechanical activation of Piezo1 drove the maturation of DCs, leading to the secretion of type I interferons as a key event in launching adaptive immunity.^[^ ^101^ ^]^ As the major effector lymphocytes for efficient killing of circulating tumor cells, NK cells performed enhanced anti‐tumor cytotoxicity under fluid shear stress.^[^ ^102^ ^]^ Further research unveiled that this mechanical‐dependent cytotoxicity might be associated with the increased production of extracellular vesicles containing tumor inhibitory molecules with high quality and quantity by NK cells after mechanical stimulation.^[^ ^103^ ^]^ A recent publication also reported that the cytoskeleton was a mechanical sensor of NK cells.^[^ ^104^ ^]^ For instance, soft substrates reduced F‐actin accumulation and talin polarization at the immunological synapse site between NK cells and target cells. This reduction thereby impaired the stable formation of the immune synapse and inhibited the cytotoxicity of NK cells.^[^ ^105^ ^]^

#### 2.2.2 Adaptive immune cells

**Figure 4:**
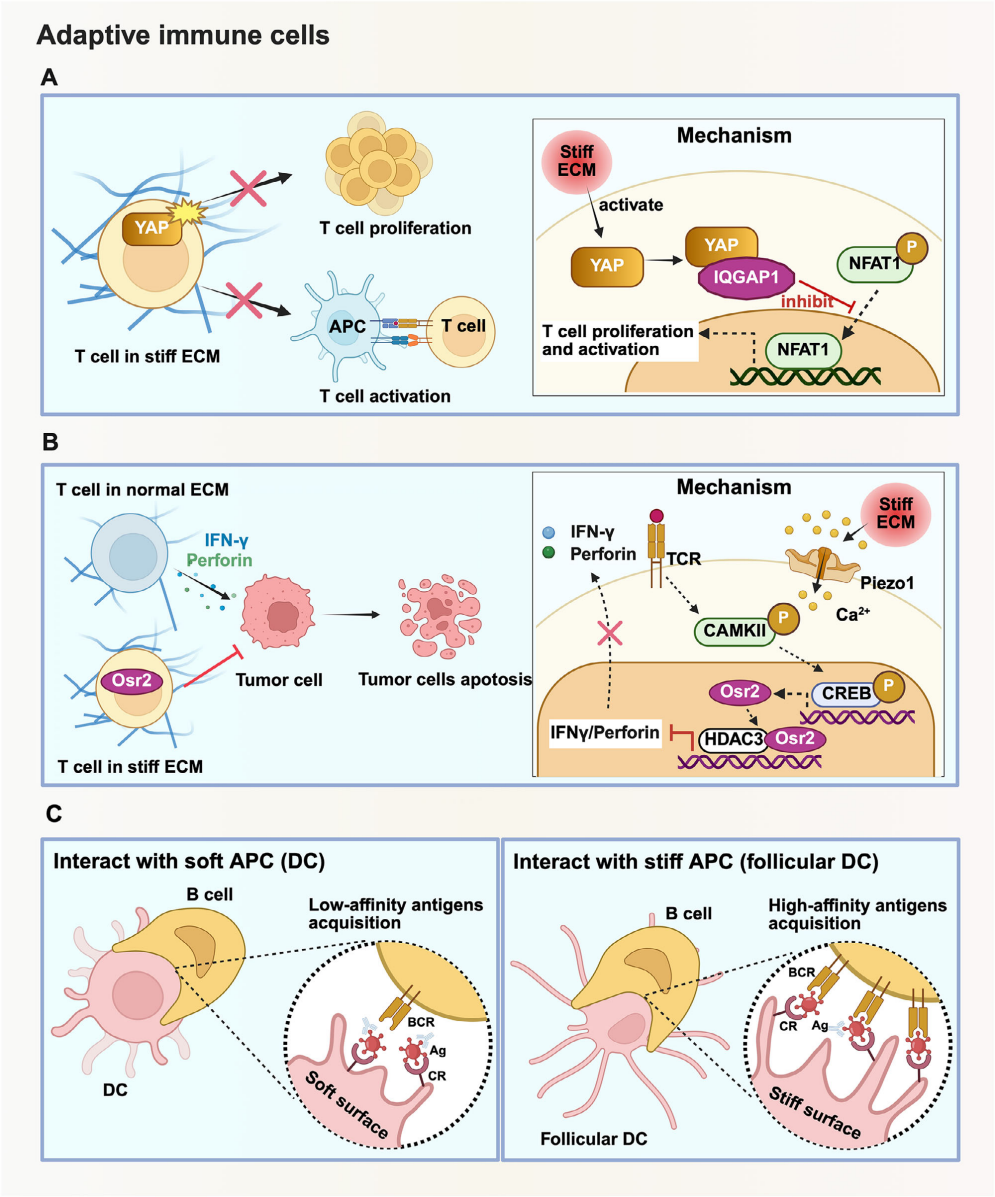
Examples of mechanical signaling regulating adaptive immune cell fate. For adaptive immune cells, mechanotransduction engages in the activation and effector function of both T cells and B cells. A) When T cells were incubated in stiff ECM, the YAP was activated in cytoplasm and translocated into nucleus, which inhibited the translocation of NFAT1 into nucleus, thus suppressing T cells proliferation and activation. B) Stiff ECM could activate Piezo1 in T cells, and regulate the secretion of IFN‐𝛾 and perforin through Osr2. C) When interacting with APCs of different stiffness, the affinity of antigens acquisition in B cells could be regulated, in which the affinity of antigens acquisition was enhanced in follicular DC (stiff APC) interaction while decreased in DC (soft APC) interaction. This figure was created with BioRender.com.

Adaptive immunity represents an evolutionarily conserved host defense system that executes highly targeted elimination of pathogens or abnormal cells including cancer cells, by recognizing specific antigens.^[^ ^106^ ^]^ It comprises two coordinated arms: T cell‐mediated cellular immunity and B cell‐mediated humoral immunity, and is also modified by mechanical cues. During their maturation and effector responses, both T cells and B cells are mechanically regulated by the mechanical property changes of the microenvironment and cell‐cell physical contacts (**Figure** **4**).^[^ ^107^ ^]^

T cells are highly mechanosensitive, largely due to the force‐dependent interaction between T cell receptors (TCRs) and peptide‐MHC complexes on APCs or target cells during immune synapse formation.^[^ ^108^, ^109^ ^]^ The mechanical properties and conformational remodeling of the cytoskeleton directly dictate the activation and effector function of T cells. Naïve CD4^+^ T cells exhibited a mechanically stiffer cytoskeleton than effector CD4^+^ T cells, leading to the formation of smaller immune synapses with APCs.^[^ ^110^ ^]^ Piezo1‐induced actin depolymerization and cytoskeletal reorganization could compromise the traction forces of cytotoxic T cells, ultimately diminishing their capacity to eliminate tumor cells.^[^ ^54^ ^]^ The phenotype and function of T cells were dynamically regulated by ECM mechanics as well.^[^ ^111^ ^]^ Interestingly, it showed that stiff ECM could suppress the proliferation, activation and effector function of T cells, in which key mechanical transcription factors such as YAP and Osr2 played a vital role.^[^ ^79^ ^]^ For instance, under the co‐stimulation of TCR signaling and biomechanical stress, the expression of transcription factor Osr2 was selectively elevated in the terminal exhaustion of tumor‐reactive cytotoxic T cells through Piezo1/Ca^2+^/CREB axis.^[^ ^112^ ^]^ Regulatory T cells (Treg cells) are also mechanically induced on stiff substrates via actomyosin contractility, highlighting the role of mechanics in immune tolerance.^[^ ^113^, ^114^ ^]^ When cultured on polyacrylamide gels with a Young's modulus of 140 kPa (stiffer matrix), the induction of Treg cells was significantly promoted compared to those cultured on gels with a modulus of 7.5 kPa. B cells have also been reported to be engaged in force‐dependent antigen extraction and presentation. The different stiffness of APCs affected the antigen uptake of B cells. Follicular dendritic cells, being stiff, enabled strong B cell pulling forces that selected for high‐affinity antigens, whereas softer dendritic cells allowed B cells to acquire antigens, including low‐affinity ones, with minimal force to facilitate broader antigen capture.^[^ ^115^ ^]^ B cell receptor (BCR) activation is also mechanically tuned through membrane lipid composition and cytoskeletal reorganization.^[^ ^66^, ^116^ ^]^ At present, the mechanobiological properties of B cells remain largely underexplored, with emerging evidence suggesting that BCR and immune synapses may function as critical mechanosensitive elements warranting further investigation in the future.

Collectively, examples of how biomechanics shapes both innate immune cells and adaptive immune cells are summarized in **Table** **1**.

**Table 1:** Examples of how biomechanics modulate immune cells.

Immune cell	Mechanical stimulus	Mechanosensors/ Mechanotransducers	Mechanoeffectors	Ref.
Macrophages	Stiff ECM	Integrin	Enhance phagocytosis	[31]
Tumor‐associated macrophages	Soft ECM elasticity	Piezo1/NF‐κB pathway	Repolarize M1 macrophages and initiate inflammatory response	[94]
Macrophages	∖	Piezo1	Enhance phagocytosis	[90]
Macrophages	Stiff ECM	Integrin	Activate M2 polarization	[92]
Neutrophils	Adhesiveness substrates	Cytoskeletons	Affect their migration and trail formation	[96]
Neutrophils	Stiff ECM	∖	Induce an anti‐inflammatory neutrophil phenotype and more anti‐inflammatory cytokine secretion	[97]
Neutrophils	Shear stress	Piezo1, cytoskeletons	Induce NETosis	[48]
Neutrophils	Stiff ECM	∖	Increase NETosis and higher secretion of pro‐inflammatory cytokines and chemokines	[98]
DCs	Stiff ECM	YAP/TAZ pathway	Enhance the proliferation, activation, and cytokine production of DCs	[47]
DCs	Stiff ECM	∖	Affect morphology and intrinsic motility patterns	[100]
DCs	Cells contact	Piezo1, cytoskeletons	Enhance the maturation of DCs and the production of type I interferons	[101]
NK cells	Fluid shear stress	∖	Enhance the clearance of circulating tumor cells	[102]
CD8^+^ T cells	∖	Piezo1	Inhibit their traction and their antitumor properties	[54]
T cells	Stiff ECM	YAP/TAZ pathway	Suppress T cells proliferation and effector function	[79]
CD8^+^ T cells	Stiff ECM	Piezo1	Aggravate exhaustion of CD8^+^ T cells and inhibit their antitumor efficacy	[112]
Treg cells	Stiff ECM	∖	Induce Treg cells proliferation	[114]
B cells	∖	Cytoskeletons	Form and stabilize immune synapses	[66]
B cells	Stiff ECM	∖	Promote selection of higher‐affinity antigen	[115]

## 3 Mechanical crosstalk in immunity

**Figure 5:**
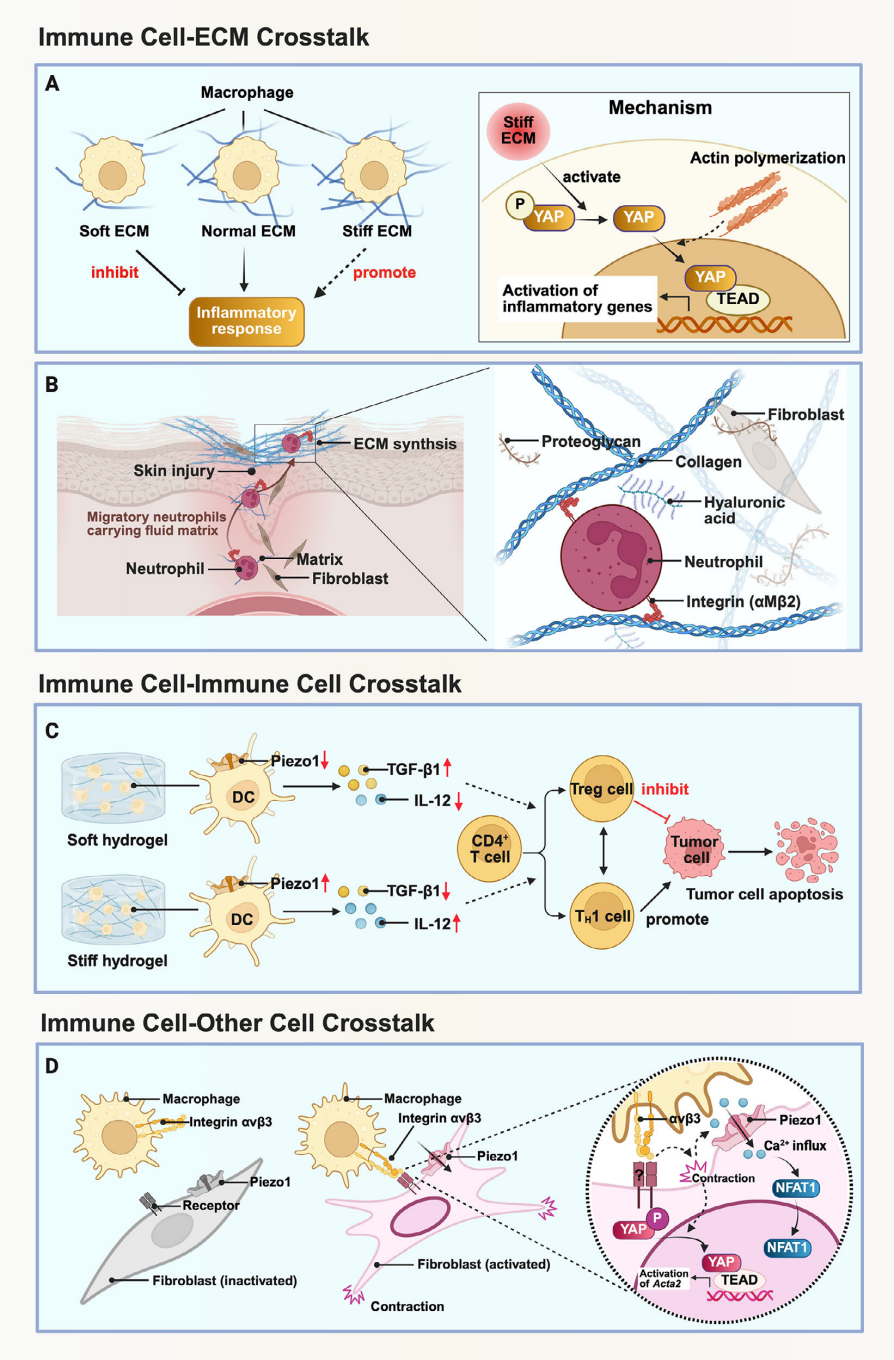
Examples of mechanical crosstalk in immunity. A) ECM shaped the behaviors of macrophages. With different stiffness of ECM, the inflammatory responses of macrophages varied. When incubated in stiff ECM, the inflammatory response of macrophages was enhanced through YAP activation and nuclear translocation. B) Neutrophils engaged in the formation of ECM. By carrying the surrounding fluid matrix with integrin αMβ2, neutrophils migrated from organs to injury sites for ECM remodeling. C) DCs incubated in different hydrogels regulated the differentiation of T cells distinctively through Piezo1 activation and cytokines secretion, thus affecting the anti‐tumor efficacy. When DCs were cultured on soft hydrogel, the activation of Piezo1 was suppressed, leading to upregulated secretion of TGF‐β1 and downregulated IL‐12 production. This microenvironmental cue promoted the differentiation of CD4^+^ T cells into Treg cells, ultimately inhibiting tumor cell apoptosis. In contrast, DCs cultured on stiff hydrogel facilitated the activation of Th1 cells, thereby enhancing anti‐tumor immune efficacy. D) Macrophages activated fibroblasts mechanically through the crosstalk between αvβ3 integrin on macrophages and Piezo1 on fibroblasts. This figure was created with BioRender.com.

Given clear evidence that mechanical stimuli regulates both innate and adaptive immune cells, it is crucial to focus on mechanical crosstalk within the immune system. Here, we summarize the mechanical crosstalk in immunity from three perspectives: the mechanical crosstalk between immune cells and ECM, the mechanical crosstalk among immune cells themselves, as well as the mechanical crosstalk between immune cells and other cells (**Figure** **5**).

### 3.1 Mechanical Crosstalk Between Immune Cells and ECM

ECM is a dynamic 3D structure surrounding cells, serving dual roles as both a mechanical scaffold and a biochemical regulator of cellular behaviors.^[^ ^117^, ^118^ ^]^ Primarily composed of fibrillar collagens, glycosaminoglycans, proteoglycans, and adhesive glycoproteins, ECM establishes continuous bidirectional communication with immune cells throughout their developmental trajectory, spanning from hematopoietic stem cell niches in embryonic tissues or bone marrow to terminal differentiation and effector phase execution in peripheral tissues. This persistent mechanobiological dialogue involves reciprocal mechanical signaling, where ECM stiffness, topography, and composition dynamically modulate immune cells fate decisions, migration patterns, and functional polarization while simultaneously receiving biochemical and mechanical feedback from the activities of immune cells.^[^ ^119^ ^]^ For example, integrin adhesion complexes mediated the adhesion and movement of immune cells by binding to ECM ligands, such as collagens, laminins and fibronectin.^[^ ^120^ ^]^ Moreover, ECM contributed to the migration of T cells, indicating that ECM had effects on the behaviors of immune cells.^[^ ^121^ ^]^ In turn, ECM can also be remodeled in respond to mechanical stimuli from immune cells.

ECM acts as critical mechanical cues which engages in dictating immune cells behaviors through mechanotransduction. Macrophages are highly mechanically sensitive cells that sense and respond to various mechanical cues from ECM. It was reported that enhanced ECM stiffness could promote the phagocytosis of macrophages through integrin‐dependent way.^[^ ^122^ ^]^ Macrophages lacking integrins β2 exhibited specific defects in the formation of phagocytic cup, an essential process in the phagocytosis of macrophages, thus the uptake of stiff cargo was suppressed. As a key mechanosensitive transcriptional coactivator, YAP also participated in the mechanical sensation in the crosstalk between macrophages and ECM. Meli et al. demonstrated that the stiffness of ECM regulated the macrophage inflammatory response through YAP signaling pathway.^[^ ^123^ ^]^ Specifically, soft ECM or depletion of YAP suppressed the inflammatory responses of macrophages, whereas stiff ECM or overexpression of YAP enhanced inflammation. In terms of mechanism, macrophages first mechanosensed the changes of ECM stiffness, which induced the polymerization of actin downstream. In response, activated YAP translocated into the nucleus and regulated the expression of inflammatory genes in the macrophages, thus mediating the mechanotransduction cascade. Additionally, it was said that the exosomes from medium stiffness substrates (GelMA hydrogels with mass concentrations of 10%) could shift macrophage polarization from M1 to M2 compared to those from soft stiffness substrates (GelMA hydrogels with mass concentrations of 5%).^[^ ^124^ ^]^ While the ECM mechanically regulates the behaviors of macrophages, it also shapes the activation and effector functions of T cells. Sapudom et al. investigated how the density and fibrillar alignment of ECM affected the behaviors of T cells using 3D collagen matrix.^[^ ^125^ ^]^ The results indicated that increased ECM stiffness induced the suppression of T cell activation, decreased cytokine production, and restricted T cell proliferation via the YAP signaling pathway. However, in pathological microenvironments of tumors and fibrosis, altered ECM stiffness disrupts immune cell homeostasis, triggering functional dysregulation and exacerbating pathological responses. It was reported that the tumor‐associated ECM was capable to generate specific tumor‐associated macrophages including M1‐like macrophages which supported inflammation and M2‐like macrophages which suppressed anti‐tumor immunity. This indicated a promising solution for anti‐tumor therapy by targeting abnormal tumor ECM.^[^ ^126^ ^]^ Another study uncovered that the T cell cytotoxicity against malignant mammary gland carcinoma cells varied upon different ECM compositions, where the function of T cells was defective in malignant mammary gland carcinoma cells/T cell cocultures on collagen IV while it was effective on vitronectin.^[^ ^127^ ^]^ All these examples suggest that the mechanics of ECM may be a novel therapeutic target for immune‐related disorders.

In vivo, immune cells dynamically interact with ECM, driving the biogenesis and remodeling of ECM. It was widely acknowledged that macrophages regulated the ECM through TGF‐β, which contributed to the promotion of fibroblast differentiation and collagen production, as well as the inhibition of metalloproteinases, an enzyme contributed to the degradation of matrix.^[^ ^120^, ^128^ ^]^ When local macrophages are dysfunctional, the mechanical properties of ECM can be altered. As reported, the inhibition of hypodermal macrophages hindered the clearance of hyaluronic acid, resulting in the swelling and stiffening of ECM.^[^ ^129^ ^]^ In addition, Witherel et al. investigated the role of macrophages in ECM assembly and found that the conditioned media from hybrid M1/M2 macrophages prompted fibroblasts to synthesize a matrix featuring thicker and disorganized fibers, whereas M2 macrophage‐conditioned media induced the formation of aligned matrix architecture with thinner fibers which was structurally preferable for ECM assembly.^[^ ^130^ ^]^ The results suggested that turning the balance toward M2 polarization could promote ECM remodeling. Neutrophils and T cells are also implicated in the remodeling of ECM. It was reported that neutrophils were able to use integrin αMβ2 to carry and transfer from the surrounding matrix across organs into wounds, which contributed to reestablish a new ECM scaffold in the wound sites as an early event of wound repair.^[^ ^131^ ^]^ Researchers also discovered that CD8^+^ T cells expressing enzyme lysyl oxidase were key contributors during the paclitaxel chemotherapy‐induced metastasis. By responding to paclitaxel, CD8^+^ T cells released lysyl oxidase in lungs, which led to the remodeling of pulmonary ECM and made the primary cancer vulnerable for metastasis.^[^ ^132^ ^]^ However, how immune cells regulate ECM on the molecular force level remains unclear.

### 3.2 Mechanical crosstalk among immune cells

Mechanical signaling has emerged as a critical mechanobiological paradigm in immunology, operating in parallel with classical chemical communication pathways.^[^ ^133^ ^]^ In addition to ECM‐mediated mechanical cues, direct cell‐cell interactions in immune systems which are mediated through mechanical forces at intercellular junctions (e.g., interfacial tension modulation) constitute another critical component of mechanical signaling crosstalk. Here, we summarize the mechanical interactions among immune cells from two perspectives: the mechanical regulation of the immune cells themselves and the mechanical regulation occurring between different immune cells.

In the mechanotransduction networks of immune cells, different mechanosensors interact and restrict mutually to regulate the behaviors of immune cells. Liu et al. found that Piezo1 played an important role during integrin‐dependent chemotaxis of T cells through mechanical “outside‐in” signaling.^[^ ^134^ ^]^ By responding to mechanical stimuli from chemokines, activated Piezo1 promoted the integrin LFA1 to recruit at the leading edge of the chemotactic T cells, eventually enhancing the migration of T cells. With the development of precise regulation at the molecular level in cells, it was said that tiny structural and component changes of mechanosensors could make a big difference in immune cells behaviors. Wang et al. discovered that localized mechanical crosstalk contributed to define the secretory events in the immune synapse.^[^ ^33^ ^]^ While the pulling forces of LFA‐1 were pivotal in secretory domains of the immune synapse, the depletion of talin disrupted such forces thus impacting the release of perforin and lytic enzymes from T cells, impairing the cytotoxicity of T cells against pathogens and tumor cells. Besides, it was suggested that mechanosensors could mutually regulate each other during mechanotransduction. While both integrins and Piezo1 performed as the mechanosensors in macrophages inflammatory responses, the knockdown of integrin CD11 enhanced the expression of Piezo1, suggesting a potential crosstalk between CD11 and Piezo1.^[^ ^135^ ^]^

The immune response constitutes a dynamic cascade of cell‐cell signaling events, with emerging evidence highlighting the mechanical crosstalk as a critical mode of intercellular communication among immune cells. As pivotal effector cells in innate immunity, neutrophils and macrophages play indispensable roles in orchestrating antiviral defense mechanisms through coordinated signaling networks. Wang et al. found that Piezo1 directed the NET formation in neutrophils, and the formation of NET then tuned the functional polarization of macrophages toward M1 type during viral infection both in vitro and in vivo.^[^ ^136^ ^]^ This suggested a close mechanical link between macrophages and neutrophils. Antigen presentation and subsequent T cell activation are important parts of cellular immunity. As one of the major APCs, DCs play critical role in anti‐tumor immunity through the induction of T cell differentiation. Piezo1 was demonstrated to mediate the of mechanotransduction DCs and direct the mutual differentiation of Th1 and Treg cells in cancer, that is, deletion of Piezo1 in DCs inhibited the generation of Th1 cells while enhanced the development of Treg cells.^[^ ^137^ ^]^ With their tailored and bio‐friendly mechanics, hydrogels have become a promising biomaterial for T cell activation. Schneck et al. developed an artificial lymph node matrix based on hydrogels which conjugating with peptide‐loaded‐MHC complex, the co‐stimulatory signal anti‐CD28, and a tethered IL‐2 as the signals for T cell activation.^[^ ^138^ ^]^ Such hydrogel platform enabled a direct CD8^+^ T cell activation in vivo, eliminating the need for ex vivo priming or expansion. Inspired by APC, Mooney et al. designed an artificial antigen‐presenting cells based on microgels, namely microscale particles of hydrogels with highly tunable surface, for T cell activation.^[^ ^139^ ^]^ By coating suitable stimulatory ligands on the surface of microgels, the activation and expansion of T cell were promoted. In addition, the development of DNA nanotechnology makes the research of cellular interactions at molecule levels possible. Du et al. designed a membrane‐anchored DNA nanojunction with distinct sizes to modulate the APC‐T cell interface at different distances.^[^ ^140^ ^]^ The results suggested that the axial distance of the contact zone was critical in T cell activation probably by modulating protein reorganization and mechanical forces which were generated from bulky molecules under compression or tension, while shortening the distance enhanced the activation of T cells. Immunological synapse is a dynamic interface formed when T cells contact APCs or target cells, and accurately directs the activation and effectors of T cells.^[^ ^141^ ^]^ It was confirmed that the APC‐T cell synaptic contact formed and stabilized via continuous cytoskeletal tension, driven by focal nucleation of actin within the plane of the synapse.^[^ ^142^ ^]^ After T cell activation, the cytoskeletons of T cells unraveled and tension decayed, causing synapse breaking as a result.

### 3.3 Mechanical crosstalk between immune cells and other cells

As vital mediators in the pathological progression, immune cells engage in the progression of diseases through mechanical crosstalk with other cells. A study uncovered that neutrophils physically interacted with breast tumor cells by applying physically interacting cells sequencing (PIC‐seq).^[^ ^143^ ^]^ The result of PIC‐seq showed that there were physical interactions between neutrophils and tumor cells which contributed to regulate the proliferative and invasive abilities of tumor cells. This suggested that tuning the physical interactions between neutrophils and tumor cells might be a promising approach in breast cancer progression. During the recognition of tumor cells or pathogens by CD8^+^ T cells, the binding of TCR and peptide‐major histocompatibility complex (pMHC), namely TCR‐pMHC can affect the accuracy of immune surveillance through mechanical crosstalk. Qin et al. demonstrated that the tightness of TCR‐pMHC binding interface on CD8^+^ T cells mechanically regulated the affinity of CD8 against MHC.^[^ ^144^ ^]^ It was said that the natural TCRs could form optimal catch bonds with pMHC which provided flexible interfaces for CD8 to bind to MHC‐α1α2 domains. In contrast, engineered high‐affinity TCRs formed rigid and tight bound interfaces with cognate pMHCs. Such tight bound interfaces blocked the force‐triggered conformational changes essential for optimal catch‐bond formation thus inhibited the binding of CD8 and MHC. The different mechanics between natural TCRs and engineered high‐affinity TCRs determined that whether CD8 could bind to MHC, implying that the mechanics of TCR regulated the accuracy of T cell's recognition. Moreover, the mechanical crosstalk between T cells and tumor cells also influences the cytotoxicity of T cells against tumor cells. It was acknowledged that the release of perforin and lyase was the key process to kill target cells in the effector stage of T cells. However, Liu et al. found that cytotoxic T lymphocytes (CTL) failed to kill soft tumor‐repopulating cells (TRC), while they effectively destroyed stiff differentiated TRC.^[^ ^145^ ^]^ Mechanically, perforin needed to interact with nonmuscle myosin heavy‐chain 9, which contributed to transmit forces to F‐actins to generate enough contractile force for perforin pore formation in TRC. However, the soft TRC couldn't provide adequate F‐actins thus failed to form perforin pore, which made CTL unable to kill soft TRC through the cytotoxicity of perforin.

Besides, there is also a close mechanical crosstalk between macrophages and fibroblasts. Ezzo et al. uncovered that the direct contact with profibrotic macrophages triggered acute contractions of fibroblasts, which was dependent on the crosstalk between integrin αvβ3 on macrophages and Piezo1 on fibroblasts.^[^ ^21^ ^]^ Upon the physical contact of fibroblasts with profibrotic macrophages, Piezo1 was activated and the cytosolic Ca^2+^ in fibroblasts was elevated within seconds, followed by the translocation of NFAT1 and YAP into nucleus, ultimately initiating the acute activation of fibroblasts. Another study revealed that fibroblasts sensed cell density through YAP pathway, which then directly regulated the expression of colony‐stimulating factor 1, a lineage‐specific growth factor in macrophages, thus increasing the number of macrophages.^[^ ^146^ ^]^

## 4 Mechanoimmunology in Health and Diseases

### 4.1 Immune Homeostasis

**Figure 6:**
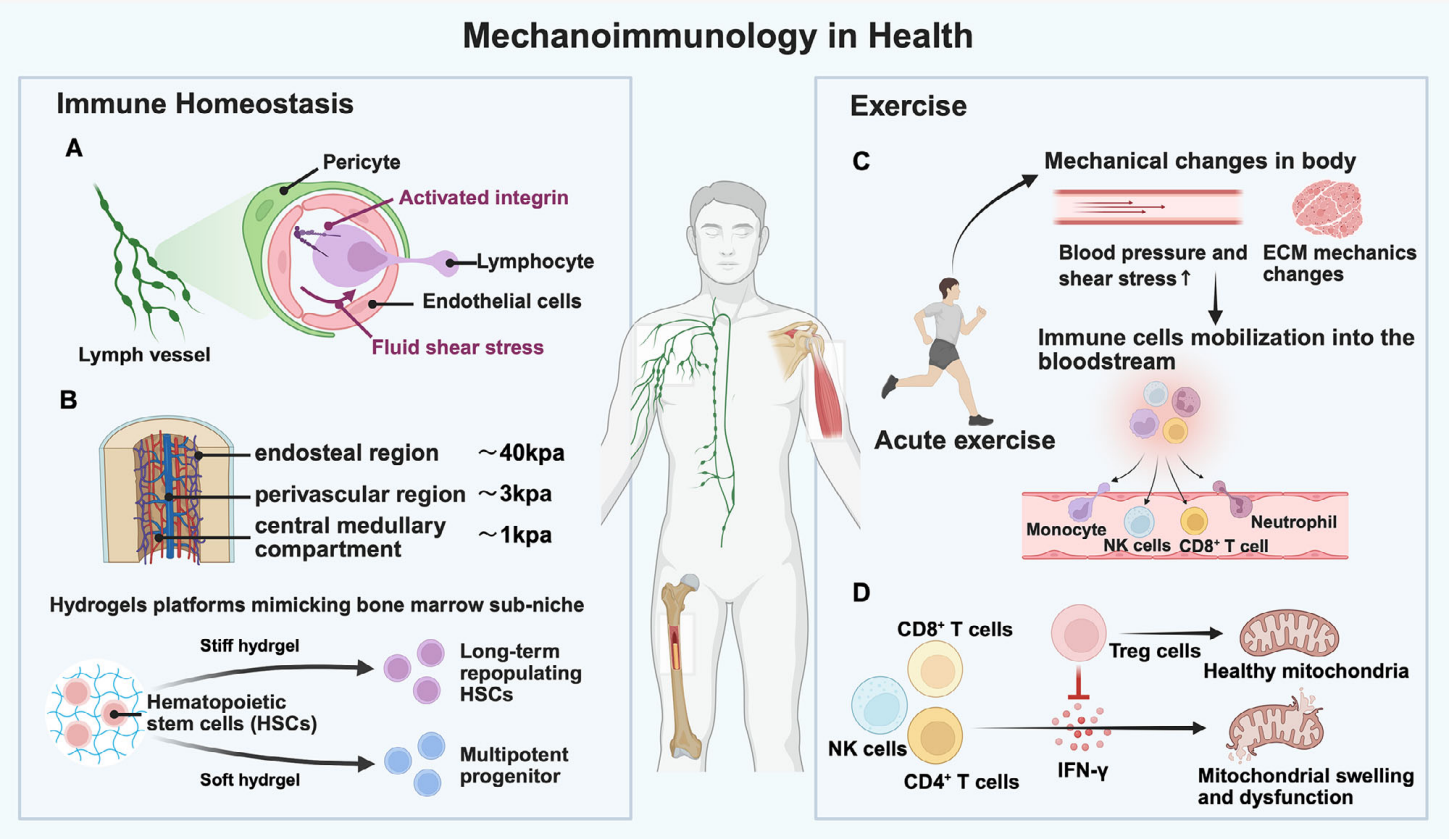
Examples of mechanoimmunology in health. A) Lymphocytes experienced fluid shear stress during transmigration across vascular or lymphatic endothelium. Activated integrins are crucial for this mechanical motility. B) The mechanical stiffness of distinct bone marrow niches regulated hematopoietic stem cell differentiation fates in hydrogel‐based models mimicking this microenvironment. C) Acute exercise induced the upregulation of immune cells. During acute exercise, there were mechanical changes in body, such as the increase of blood pressure and shear stress, as well as ECM mechanics changes. By responding to these mechanical changes, immune cells mobilized into the bloodstream. D) During exercise, Treg cells in skeletal muscles could inhibit IFN‐γ from NK cells, CD4^+^ T cells and CD8^+^ T cells to maintain the mitochondrial metabolism. This figure was created with BioRender.com.

Mechanotransduction plays fundamental roles in maintaining immune homeostasis under normal physiological conditions. In the physiological bone marrow microenvironment, matrix stiffness and biomechanical cues play essential roles in regulating myeloid immune cell development. The marrow niche displays heterogeneous stiffness, with the endosteal region being relatively stiff (∼40 kPa), the perivascular region moderately soft (∼3 kPa), and the central medullary compartment displaying the lowest stiffness at ≈1 kPa.^[^ ^147^ ^]^ Such mechanical gradients directly influence the fate of hematopoietic stem and progenitor cells (HSPCs). By using polyacrylamide hydrogels platforms mimicking bone marrow sub‐niche stiffness (2, 8, or 35 kPa), recent study demonstrated that higher stiffness helped maintain long‐term repopulating hematopoietic stem cells, whereas lower stiffness favored multipotent progenitor viability, highlighting the role of mechanical heterogeneity in modulating the differentiation of HSPCs toward myeloid and lymphoid lineages (**Figure** **6**B).^[^ ^148^ ^]^ As a primary lymphoid organ, the thymus serves as a pivotal site for T cell development and maturation.^[^ ^149^ ^]^ The thymus provides a specialized microenvironment in which thymic epithelial cells (TECs) and an ECM‐rich 3D scaffold orchestrating the intrathymic maturation and selection of T cells.^[^ ^150^ ^]^ Hong et al. used assays including biomembrane force probe to reveal that upon encountering self pMHC ligands during negative selection, thymocytes exerted forces on their TCR and CD8 co‐receptors, forming cooperative TCR‐pMHC‐CD8 “catch bonds”, which was a specialized type of bond whose lifetime lengthened under applied force.^[^ ^151^ ^]^ In contrast, positive‐selection ligands induce only transient “slip bonds” between TCR‐pMHC or pMHC‐CD8. Crucially, the catch bonds were not intrinsic to either the TCR‐pMHC or pMHC‐CD8 arm of the trans heterodimer. Rather, they resulted from the coupling of the extracellular pMHC‐CD8 interaction to the intracellular binding of CD8 to TCR‐CD3. This coupling, mediated by associated kinases, formed a cis heterodimer capable of inside‐out mechanotransduction signaling that uniquely amplified negative‐selection signals. In addition, Asnaghi et al. engineered a 3D scaffold derived from decellularized thymic ECM that preserved key features of the native thymic niche.^[^ ^150^ ^]^ This scaffold supported both in vitro and in vivo thymopoiesis when seeded with TECs, enabling sustained T cell development and culture viability. Together, these findings establish the integrated mechanical microenvironment of the thymus that regulates T cell proliferation, TCR rearrangement, and both positive and negative section. Beyond matrix‐ and cell‐derived mechanical signals, fluid shear stress represents a unique physical cue that encountered by lymphocytes when lymphocytes travel through blood and lymphatic vessels. During physiological circulation, shear stress promoted transendothelial migration of lymphocytes in an integrin‐dependent manner, a process that required cytoskeleton and chemokine signaling (Figure 6A).^[^ ^152^ ^]^ High‐affinity LFA‐1 integrin under shear forces supported lymphocyte crawling and increased the formation of adhesive filopodia that penetrated the endothelium, thereby facilitating the transmigration of lymphocytes.^[^ ^153^ ^]^ Additionally, shear stress modulated the directional migration of T cells through the specific integrin–ligand interactions: engagement of LFA‐1 with ICAM‐1 directed T cells to migrate upstream against the flow, whereas engagement of VLA‐4 with VCAM‐1 promoted downstream migration.^[^ ^154^ ^]^ These studies establish that fluid shear stress is a key regulator of integrin activation and lymphocyte transmigration.

### 4.2 Exercise

Exercise is widely recognized for strengthening immune competence and reducing disease vulnerability. It fights chronic inflammation associated with heart disease, cancer, and diabetes via immunomodulation.^[^ ^155^ ^]^ Strong evidence has shown that higher‐intensity exercise contributes to lower all‐cause mortality and potentially prevents half of cancer deaths.^[^ ^132^, ^133^ ^]^ Acute exercise induces biphasic lymphocytosis, with NK cells increasing significantly during the initial response (Figure 6A).^[^ ^156^ ^]^ To explore the underlying mechanism, it was reported that dynamic exercise led to an increase in circulating ICAM‐1 which might shed from the surface of lymphocytes.^[^ ^157^ ^]^ This suggested that mechanical forces may play a potential role in exercise‐related immune modulation. Indeed, studies have shown that during acute physical activity, blood pressure and shear stress of blood flow increase, and the mechanics of ECM in skeletal muscle also undergoes changes (Figure 6C).^[^ ^158^ ^]^ As muscles mediate movement and immune metabolism in physical activity, it is essential to investigate the potential interplay between skeletal muscle activity and mechanoimmunology. During skeletal muscle contraction, mitochondrial oxidation represents a typical metabolic adaptation to endurance exercise.^[^ ^159^ ^]^ Interestingly, Langston et al. reported a previously unknown role for Treg cells in exercise adaptation and demonstrated that exercise rapidly induced the expansion of Treg cells in the muscle.^[^ ^160^ ^]^ During exercise, Treg cells contributed to alleviate the overload of proinflammatory protein IFN‐γ which was secreted from CD4^+^ T cells, CD8^+^ T cells and NK cells, thus avoiding the mitochondrial metabolic disorder caused by IFN‐γ (Figure 6D). In muscle injury sites, uneven mechanical stress distribution can lead to an increase in pro‐inflammatory macrophages, which is detrimental to the repair of muscle injury. Wang et al. designed a modified biomaterials in order to optimize the mechanical stress distribution in injured muscles.^[^ ^161^ ^]^ Without uneven mechanical stimuli, the pro‐inflammatory macrophages reduced which was conducive to the repair of muscle injury. Moreover, a study carried by the National Aeronautics and Space Administration showed that upon return to Earth after a one‐year mission on the International Space Station, the astronaut exhibited elevated levels of proinflammatory classical monocytes (CD14⁺, CD16^−^) in the periphery compared to pre‐flight levels.^[^ ^162^ ^]^ In the future, more mechanisms on how mechanoimmunology influences exercise deserved to be discovered.

### 4.3 Inflammation and Infection

**Figure 7:**
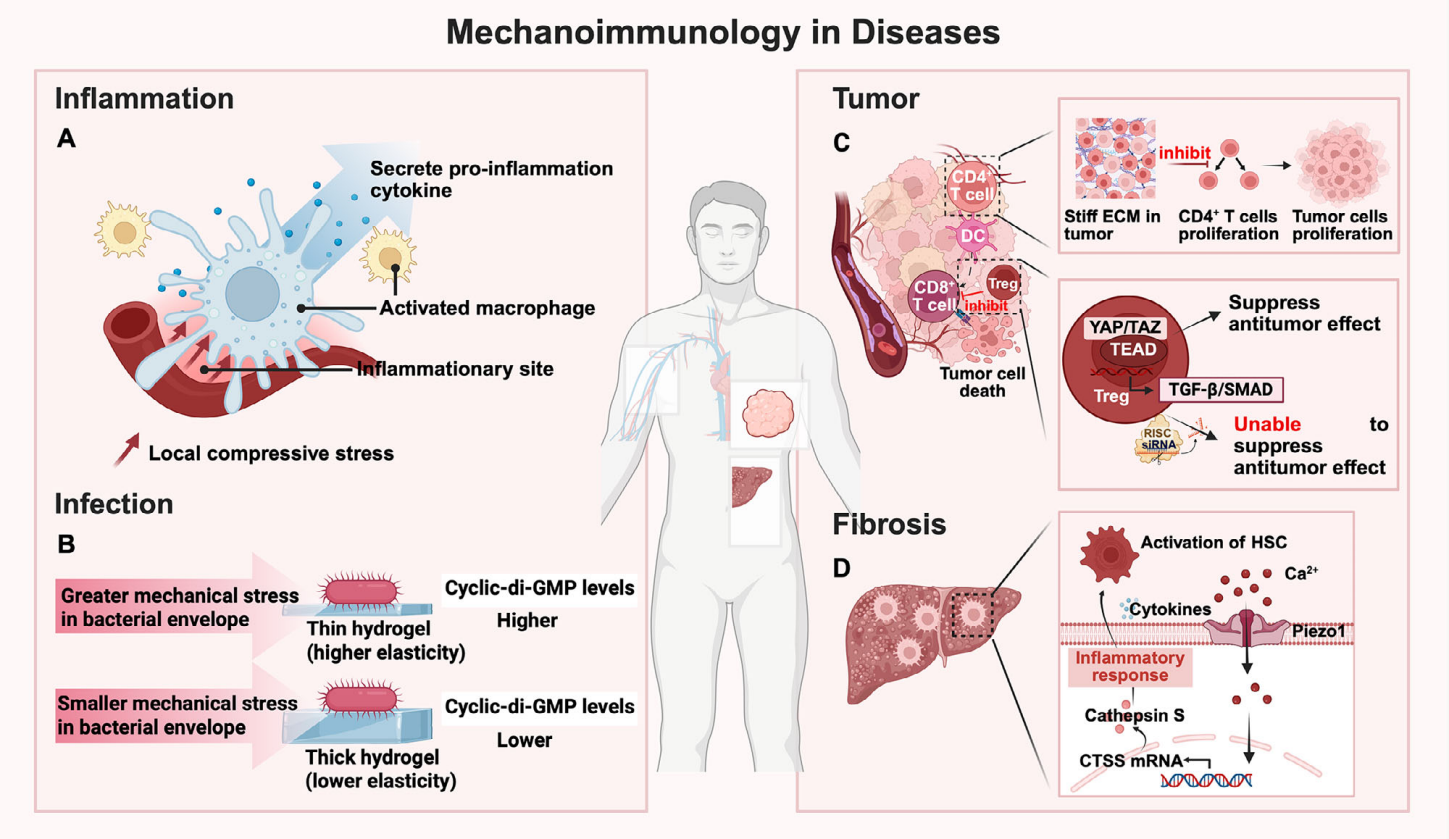
Examples of mechanoimmunology in diseases. A) Local compressive stress from plasma leakage and interstitial fluid accumulation at inflammatory sites activated macrophages, eliciting the secretion of robust pro‐inflammatory cytokine. B) Bacteria modulated their biological responses dependent on different substrate stiffness, exhibiting higher cyclic‐di‐GMP activity on thin hydrogel that promoted biofilm formation. C) Stiff ECM in tumor inhibited CD4^+^ T cells proliferation, thus weakening the killing efficacy against tumor cells and aggravating tumor growth. After silencing YAP in Treg cells, the anti‐tumor suppression was limited because of the dysfunction of Treg cells, suggesting that YAP might be an antitumor immunotherapeutic target in Treg cells. D) In fibrosis, macrophages sensed the fibrotic substrate through Piezo1, following with an increased Ca^2+^ influx and regulating the secretion of cathepsin S (CTSS). As a result, the hepatic stellate cells (HSCs) were activated which further aggravate the process of fibrosis. This figure was created with BioRender.com.

Mechanotransduction also critically shapes inflammatory processes. Inflammatory responses are frequently accompanied by plasma leakage and interstitial fluid accumulation, which generate local compressive stress and alter interstitial fluid pressure. These biomechanical changes are not passive byproducts but actively shape immune cell function. Studies showed that compressive loading and shear stress enhanced proinflammatory gene expression and cytokine secretion in monocytes and macrophages, highlighting a direct mechanosensitive pathway that promoted inflammatory activation (**Figure** **7**A).^[^ ^163^, ^164^ ^]^ Also, inflammatory stimuli modulated interstitial fluid pressure by altering integrin‐collagen binding, thereby promoting edema formation. Further inhibition of 𝑎11 integrins modified interstitial fluid pressure and the extent of inflammatory swelling.^[^ ^165^ ^]^ Moreover, impaired lymphatic drainage during inflammation exacerbates fluid retention and increases tissue pressure, establishing a feedback loop in which mechanical loading amplifies immune dysfunction.^[^ ^166^ ^]^ Reviews of lymphatic biology emphasized that impaired lymphatic clearance led to accumulation of pro‐inflammatory cytokines locally and altered immune cell trafficking.^[^ ^166^ ^]^ Studies revealed that lymphatic dysfunction was causal in amplifying inflammation and reshaping immunity, while restoring lymph flow was anti‐inflammatory. In the lung, boosting lymphatic drainage with VEGF‐C156S, a ligand of VEGFR‐3, accelerated the clearance of inflammatory cells and edema in sepsis‐induced acute respiratory distress syndrome. And pharmacologic blockade of VEGFR‐3/CCL21 abrogated this effect of VEGF‐C156S, establishing mechanism‐based specificity. In the central nervous system, prophylactic VEGF‐C enhanced the function of meningeal lymphatic vessels and reduced neuroinflammation, resulting in the improvement of post‐stroke outcomes, thus linking fluid pressure and drainage capacity to immune tone in the brain.^[^ ^167^ ^]^

In the context of infection, both host and pathogen exploit mechanical pathways. For instance, influenza virus was reported to activate Cdc42‐mediated signaling to stimulate filopodia formation, resulting in the enhancement of viral endocytosis. However, host cells counteracted this with cortactin phosphorylation, which limited filopodia and curbed viral entry.^[^ ^168^ ^]^ Quantum dot‐based single‐virus tracking of influenza A virus further revealed that influenza A virus was able to exploit distinct pathways to reach the microtubule network, involving either actin‐dependent transport or direct microtubule engagement. This finding revealed multiple, parallel routes of virus for intracellular trafficking, underscoring the flexibility of influenza A in navigating host cytoskeletal systems to ensure efficient infection.^[^ ^169^ ^]^ Furthermore, bacteria can mechanically remodel their microenvironment. Bacterial populations actively remodel their microenvironment through mechanical strategies that facilitate surface colonization, biofilm maturation, and host‐tissue disruption. For example, bacterial biofilms generated growth‐induced mechanical forces that were strong enough to deform soft substrates and buckle epithelial layers, revealing that biofilm expansion alone could physically disrupt host tissues and contribute to pathogenesis.^[^ ^170^ ^]^ Besides, the accumulation and growth of the biofilm‐forming pathogen *Pseudomonas aeruginosa* on surfaces were strongly influenced by substrate mechanics. Softer or stiffer surfaces altered bacterial adhesion and colony architecture by regulating intracellular cyclic‐di‐GMP signaling. Disruption of this pathway impaired surface‐associated growth, further highlighting that mechanical cues were transduced into biochemical signals to control biofilm development and persistence (Figure 7B).^[^ ^171^ ^]^ Bacterial colonies also tune their own rheological and adhesive properties in response to substrate mechanics and external stress. Such adaptive remodeling enhances colony stability and resilience, demonstrating that bacteria actively tune their collective mechanics to optimize survival and biofilm persistence under varying physical conditions.^[^ ^172^ ^]^ Finally, mechanical forces generated during bacterial growth could trigger a reorientation cascade within biofilms. As bacterial cells proliferated, compressive stresses drove local realignments, which propagated collectively to form ordered, large‐scale patterns, illustrating that mechanical remodeling was a strategy used by bacteria to engineer their niche.^[^ ^173^ ^]^

### 4.4 Tumor

**Figure 8:**
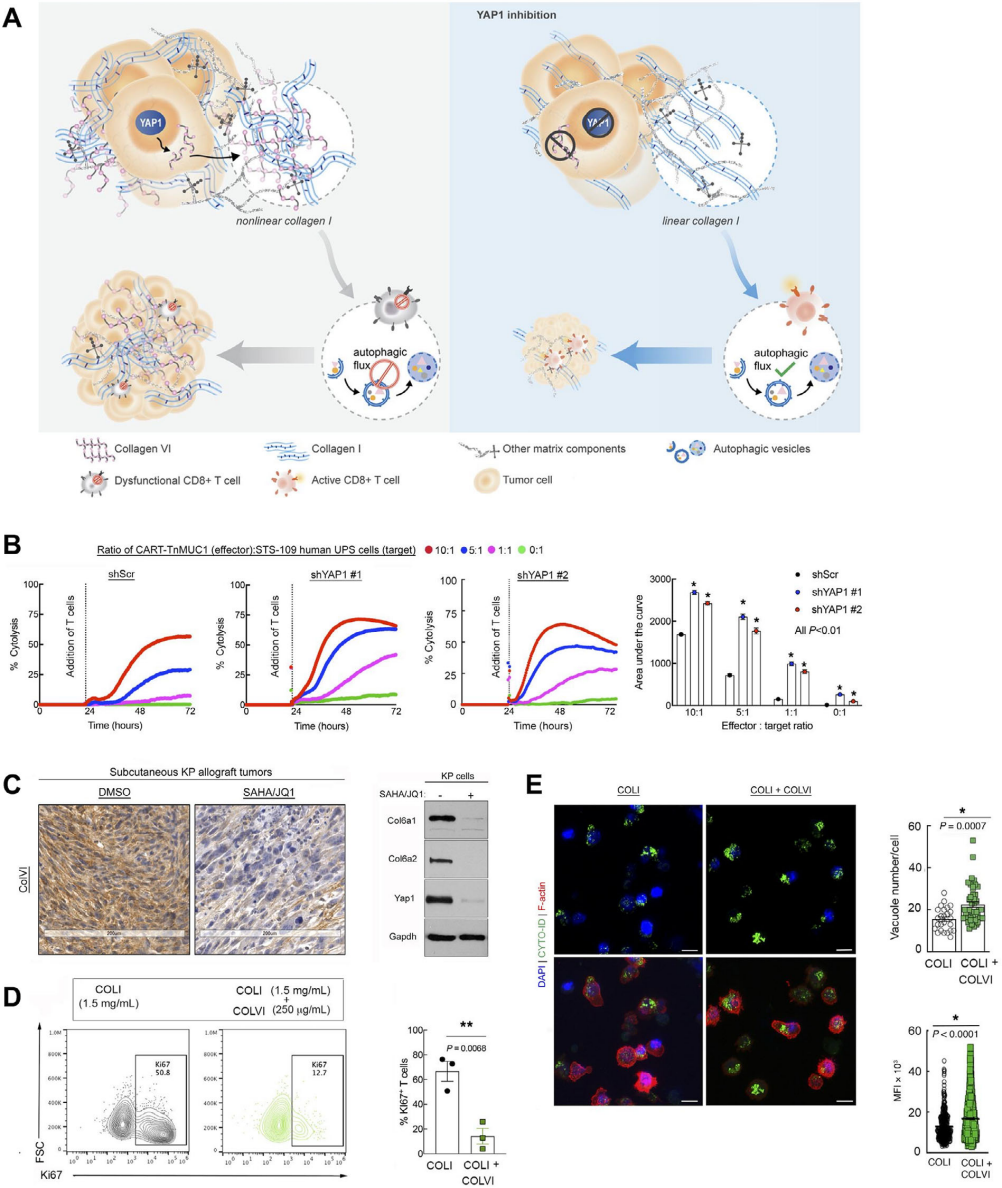
YAP mediated ECM remodeling in the tumor microenvironment, thereby influencing the cytotoxicity of CD8^+^ T cells. A) Graphic illustration of how YAP‐mediated tumor microenvironment mechanically regulated the cytotoxicity of CD8^+^ T cells. B) Suppression of YAP in UPS cells enhanced the cytotoxicity of CD8^+^ T cells and accelerated the cytolysis of UPS cells. Average longitudinal cytolysis of shScr or shYAP1 human STS‐109 UPS cells during coculture with CART‐TnMUC1 cells from 3 independent human donors. C) Intrinsic YAP of tumor cells was shown to be involved in regulating the ColVI deposition in the TME. Representative images of ColVI IHC in KP tumor‐bearing mice treated with SAHA plus JQ1 (an antitumor therapy) or vehicle control for 20 days. Representative immunoblot of KP cells treated with SAHA plus JQ1 or vehicle control for 48 h. D) The presence of ColVI damaged the ColI‐mediated CD8^+^ T cell cytotoxicity. Representative flow cytometry plots and quantification of Ki67 in activated human CD8^+^CD44^+^ T cells after incubation on hydrogels containing purified COLI with or without purified COLVI. E) ColVI suppressed CD8^+^ cell autophagy. Visualization and quantification of autophagosomes in CD8^+^ T cells cultured on hydrogels containing purified COLI with or without purified COLVI. Reproduced under terms of the CC‐BY license.^[^ ^193^ ^]^ Copyright 2024, The Authors, published by the American Society for Clinical Investigation.

Immune cells and tumors are always in the dynamic balance of the “offensive and defensive” game. On one hand, tumors reshape the immune microenvironment through a variety of mechanisms. On the other hand, immune cells modulate tumor development by maintaining the balance between immune equilibrium (e.g., DC cells, CTLs and NK cells) and immune evasion (e.g., Treg cells).^[^ ^174^, ^175^, ^176^ ^]^ As a key survival strategy promoting tumor growth and progression, immune suppression is mainly characterized by the decline of immune defense and immune surveillance.^[^ ^177^, ^178^ ^]^ And how to break through immune suppression has always been a research hotspot and difficulty in anti‐tumor therapy. Studies have found that the occurrence of tumor immunosuppression is closely related to the communication between immune cells and the signals in the tumor microenvironment (TME).^[^ ^179^ ^]^ For instance, researchers found that there was a high expression of programmed cell death‐ligand1 (PD‐L1) on tumor cells which could bind to programmed cell death‐1 (PD‐1) on T cells, transmitting a strong inhibitory signal and thereby shutting down the anti‐tumor activity of T cells.^[^ ^180^ ^]^ Based on this, the anti‐PD‐1 therapy was developed and obtained satisfactory therapeutic effect in some cancer's treatment.^[^ ^181^ ^]^ Besides PD‐1, other immune checkpoint proteins were also discovered.^[^ ^182^ ^]^ Luo et al. discovered that CD300ld on the surface of neutrophils was a crucial immune suppressor for tumor‐related immunosuppression.^[^ ^183^ ^]^ The results showed that the inhibition of CD300ld activity suppressed tumor development and exhibited a synergetic effect with anti‐PD‐1 therapy. Since a large number of studies have revealed that cells sense the biophysical properties in TME through mechanosensors such as integrins and Piezo1, it has been suggested that the regulation of mechano‐responsiveness in immune cells may represent a promising strategy for anti‐tumor therapy.^[^ ^184^, ^185^ ^]^ For instance, the inhibition of Piezo1 in myeloid cells could suppress tumor development.^[^ ^186^ ^]^ The YAP/TAZ signaling pathway performed as an emerging mechanotransducer in elaborating tumor cells‐immune cells interactions.^[^ ^187^ ^]^ Ni et al. reported that YAP was highly expressed in Treg cells and contributed to enhance the function of Treg cells (Figure 7C).^[^ ^188^ ^]^ Furthermore, the absence of YAP resulted in the dysfunction of Treg cells, impairing their abilities of either suppressing antitumor immunity or promoting tumor growth in mice, suggesting that YAP was potentially an anti‐tumor immunotherapeutic target in Treg cells. By utilizing the different mechanical sensitivities of malignant T cells and cytotoxic T cells, moderate inhibition of YAP could stiffen malignant T cells but prevent CTLs from being killed, therefore CTLs killed malignant cells without autolysis and improved the therapeutic efficacy against T cell leukemia.^[^ ^189^ ^]^ While ECM stiffening drove tumor progression by promoting cancer cell invasion, angiogenesis, and immunosuppression, increasing evidence suggested that the mechanical crosstalk between ECM and T cells within tumor microenvironment had shown great potential in anti‐tumor therapy.^[^ ^85^, ^190^, ^191^ ^]^ Kuczek et al. discovered that high‐density ECM significantly reduced the proliferation of T cells as well as led to a higher ratio of CD4^+^ (Treg cells) to CD8^+^ T cells (CTLs) compared to the low‐density ECM, aggravating the development of tumor as a result (Figure 7C).^[^ ^192^ ^]^ In another study, CD8^+^ T cells were modulated by the ECM composition where the collagen VI induced CD8^+^ T cells dysfunction and immune evasion while collagen I enhanced the anti‐tumor ability of CD8^+^ T cells (**Figure** **8**).^[^ ^193^ ^]^ Moreover, the results showed that YAP played a crucial role in the deposition of ColVI, a pro‐tumor matrix protein in the ECM of undifferentiated pleomorphic sarcoma. This suggested that YAP targeting may be a promising strategy in the antitumor therapy against undifferentiated pleomorphic sarcoma. Since DC showed distinguishing behaviors when embedded in soft and stiff hydrogels, integrating the mechanical sensitivity of DC with DC vaccine therapy may provide a innovate direction for anti‐tumor therapy.^[^ ^47^, ^194^ ^]^ DC vaccines are autologous immune therapies where patient‐derived DCs are loaded ex vivo with tumor antigens (also called neoantigen), then activated to mature state and injected to stimulate tumor‐specific T‐cell responses against tumor cells.^[^ ^195^, ^196^ ^]^ During the process of DC activation ex vivo, constructing a suitable ECM platform by regulating matrix stiffness and modifying cytokines may enhance DC maturation and mobility.

### 4.5 Fibrosis

Fibrosis is an outcome of a dysregulated tissue repair characterized by the aberrant proliferation of fibrous connective tissue and the excessive accumulation of ECM. Mediated by the concerted function of fibroblasts and innate immune cells in response to tissue perturbation, fibrosis is frequently associated with chronic pathophysiological states, including tumor, aging, and persistent infectious diseases.^[^ ^197^, ^198^ ^]^ While fibroblasts are recognized as the main effector cells in fibrosis, immune cells such as macrophages are reported to have a close crosstalk with fibroblasts, thus influencing the progression of fibrosis.^[^ ^199^, ^200^ ^]^ For instance, Cohen et al. demonstrated that a pro‐inflammatory fibroblast population (named CXCL‐iFibro) could attract and switch macrophages into inflammatory types, while macrophages promoted CXCL‐iFibro to change into ECM‐secreting myofibroblasts, promoting fibrosis in chronic kidney disease.^[^ ^201^ ^]^ Besides, immune cells could relieve the burden of fibrosis by degrading the excessive accumulation of ECM collagen. Ma et al. found that M1 macrophages significantly relieved liver fibrosis and were effective for cytotherapy in experimental liver fibrosis.^[^ ^202^ ^]^ By recruiting and activating endogenous macrophages and NK cells, the ECM collagen was largely degraded and hepatic stellate cells were induced to apoptosis. This suggested that a management of immune cells may become a promising direction for anti‐fibrosis therapy.^[^ ^203^ ^]^ Moreover, since there is stiffer ECM in fibrosis, immune cells are often activated mechanically and producing inflammatory cytokines, thus aggravating the progression of fibrosis. For instance, it was said that macrophages contributed to produce the major pro‐fibrogenic cytokine TGF‐β and initiate myofibroblast activation during the kidney fibrosis, while the suppression of specific profibrotic macrophage subset could relieve fibrosis.^[^ ^204^ ^]^ Another study showed that neutrophils might enhance the progression of fibrosis by TGF‐β secretion as well.^[^ ^205^ ^]^ Interestingly, Luo et al. discovered that the intensity of TGF‐β signaling contributed to determine the differentiation of M2a and M2c subtypes in renal fibrosis.^[^ ^206^ ^]^ In renal fibrosis, M2a macrophages exacerbated the progression of renal fibrosis by transforming into myofibroblasts; conversely, M2c macrophages exerted anti‐inflammatory effects and alleviated the degree of fibrosis. The results demonstrated that excessive TGF‐β induced M2a macrophages, while moderate TGF‐β promoted M2c macrophages. Based on this finding, researchers successfully modulated the expression of TGF‐β in macrophages via targeted nanotechnology to achieve a balance between M2a and M2c macrophages, ultimately realizing the renal fibrosis resolution. In addition, the overexpression of TGF‐β was regarded as a prominent inducer of excess immune suppression in fibrosis since it could aggravate the profibrotic progression.^[^ ^207^ ^]^ Therefore, TGF‐β could serve as a potential target in immune suppression‐related fibrosis. Zhang et al. discovered that the deficiency of IL‐24 in mice could significantly decreased TGF‐β production in the lung, leading to less M2 macrophage infiltration and relieving the lung fibrosis.^[^ ^208^ ^]^ As an acknowledged mechanosensor on macrophages, Piezo1 was detected upregulated in human and murine fibrotic liver samples.^[^ ^209^ ^]^ Mechanically, the activation of Piezo1 enhanced macrophages inflammatory response and increased the secretion of cathepsin S (CTSS), an important lysosomal protease in ECM remodeling), thus promoting fibrosis development (Figure 7D). In vivo, the results suggested that knockout of Piezo1 could inhibit the progression of liver fibrosis.

## 5 Conclusions and perspectives

Immune cells are regulated and controlled under various cellular signals throughout their whole life. While previous studies focusing on how chemical signals contribute to immune response, we still lack an understanding of how mechanical forces regulate immune cells since the life of immune cells is characterized by a series of physical processes. Emerging evidence suggests that the dynamic interplay between mechanical forces and immune cells provides a novel intercellular communication for immunology. Mechanically, immune cells hold inherit mechanical sensitivity which conduct mechanotransduction through various mechanosensors, including integrin, Piezo1, TRPV4 and mechanotranducers including YAP/TAZ. Under diverse mechanical stimuli such as ECM stiffening and compressive force, both innate immune cells and adaptive immune cells are activated through mechanotransduction cascades and trigger downstream reactions afterward to regulate immune response. Moreover, the mechanical crosstalk between immune cells and ECM or other cells provides a promising strategy to modulate immune response by tuning the mechanical sensitivities of immune cells. While the unbalance of such mechanical crosstalk may result in immune‐related diseases, future research on how to integrate mechanoimmunology with other therapies is encouraged. Targeting mechanosensitive proteins and pathways in immune cells represents a promising therapeutic frontier, yet current mechano‐targeting agents (e.g., YAP/TAZ inhibitors) face significant clinical challenges including systemic toxicity due to poor tissue specificity. To overcome this, stimuli‐responsive “smart delivery systems” such as matrix stiffness‐activated nanocarriers may provide spatiotemporal control of mechano‐targeting agents. Besides, remodeling the mechanics of TME is critical to guarantee optimal immune cell function. While TME of solid tumors is often highly fibrotic, designing functional biomaterials to degrade the matrix stiffness is conducive to the anti‐tumor effect of immune cells and reduces the immunosuppressive effect. It can also be combined with oxygen therapy and anti‐PD‐1 therapy to achieve a combined anti‐tumor effect. However, current research mainly focuses on the mechanical‐stimulated immune response at the 2D matrix level. Considering the complex extracellular environment in vivo, it is necessary to construct 3D matrix or even organoid models to more accurately simulate the mechanical response of immune cells under real circumstances.

## Conflict of Interest

The authors declare no conflict of interest.

## Data Availability

The data are available upon request from the corresponding authors.

## References

[1] J. Ochando , W. J. M. Mulder , J. C. Madsen , M. G. Netea , R. Duivenvoorden , Nat. Rev. Nephrol. 2023, 19, 23. 36253509 10.1038/s41581-022-00633-5 PMC9575643

[2] R. L. Sabado , S. Balan , N. Bhardwaj , Cell Res. 2017, 27, 74. 28025976 10.1038/cr.2016.157 PMC5223236

[3] B. Pulendran , M. M. Davis , Science 2020, 369. 10.1126/science.aay4014 PMC7872131 32973003

[4] R. Medzhitov , Nature 2007, 449, 819. 17943118 10.1038/nature06246

[5] J. Su , Y. Song , Z. Zhu , X. Huang , J. Fan , J. Qiao , F. Mao , Signal Transduct. Target Ther. 2024, 9, 196. 39107318 10.1038/s41392-024-01888-z PMC11382761

[6] G. Altan‐Bonnet , R. Mukherjee , Nat. Rev. Immunol. 2019, 19, 205. 30770905 10.1038/s41577-019-0131-x PMC8126146

[7] C. Dong , Annu. Rev. Immunol. 2021, 39, 51. 33428453 10.1146/annurev-immunol-061020-053702

[8] A. S. Mirchandani , M. A. Sanchez‐Garcia , S. R. Walmsley , Nat. Rev. Immunol. 2025, 25, 161. 39349943 10.1038/s41577-024-01087-5

[9] C. Zhu , W. Chen , J. Lou , W. Rittase , K. Li , Nat. Immunol. 2019, 20, 1269. 31534240 10.1038/s41590-019-0491-1 PMC7592628

[10] A. Akhmanova , L. C. Kapitein , Nat. Rev. Mol. Cell Biol. 2022, 23, 541. 35383336 10.1038/s41580-022-00473-y

[11] S. Phuyal , P. Romani , S. Dupont , H. Farhan , Trends Cell Biol. 2023, 33, 1049. 37236902 10.1016/j.tcb.2023.05.001

[12] T. M. J. Evers , L. J. Holt , S. Alberti , A. Mashaghi , Nat. Metab. 2021, 3, 456. 33875882 10.1038/s42255-021-00384-w PMC8863344

[13] M. Bagnat , B. Daga , S. Di Talia , Annu. Rev. Cell Dev. Biol. 2022, 38, 375. 35804476 10.1146/annurev-cellbio-120320-033250 PMC9675319

[14] C. K. Cheng , N. Wang , L. Wang , Y. Huang , Circ. Res. 2025, 136, 752. 40146803 10.1161/CIRCRESAHA.124.325685 PMC11949231

[15] J. A. Linke , L. L. Munn , R. K. Jain , Nat. Rev. Cancer 2024, 24, 768. 39390249 10.1038/s41568-024-00745-z PMC12967324

[16] M. Chen , Y. Zhang , P. Zhou , X. Liu , H. Zhao , X. Zhou , Q. Gu , B. Li , X. Zhu , Q. Shi , Bioact. Mater. 2020, 5, 880. 32637751 10.1016/j.bioactmat.2020.05.004 PMC7332470

[17] S. M. Swain , R. A. Liddle , J. Biol. Chem. 2021, 296, 100171. 33298523 10.1074/jbc.RA120.015059 PMC7948745

[18] C. Li , S. Qiu , X. Liu , F. Guo , J. Zhai , Z. Li , L. Deng , L. Ge , H. Qian , L. Yang , B. Xu , Signal Transduct Target Ther. 2023, 8, 247. 37369642 10.1038/s41392-023-01453-0 PMC10300038

[19] T. Gao , N. A. Maskalenko , S. Kabir , K. S. Campbell , J. Wu , Cell Rep. 2025, 44, 115607. 40310722 10.1016/j.celrep.2025.115607 PMC12237089

[20] X. Di , X. Gao , L. Peng , J. Ai , X. Jin , S. Qi , H. Li , K. Wang , D. Luo , Signal Transduct. Target Ther. 2023, 8, 282. 37518181 10.1038/s41392-023-01501-9 PMC10387486

[21] M. Ezzo , K. Spindler , J. B. Wang , D. Lee , G. Pecoraro , J. Cowen , P. Pakshir , B. Hinz , Sci. Adv. 2024, 10, adp4726. 10.1126/sciadv.adp4726 PMC11498225 39441936

[22] S. Liu , Y. Li , Y. Hong , M. Wang , H. Zhang , J. Ma , K. Qu , G. Huang , T. J. Lu , Adv. Drug Deliv. Rev. 2023, 194, 114722. 36738968 10.1016/j.addr.2023.114722

[23] J. Tao , Y. Li , D. K. Vig , S. X. Sun , Rep. Prog. Phys. 2017, 80, 036601. 28129208 10.1088/1361-6633/aa5282 PMC5518794

[24] G. Halder , S. Dupont , S. Piccolo , Nat. Rev. Mol. Cell Biol. 2012, 13, 591. 22895435 10.1038/nrm3416

[25] G. F. G. , R. E. , Science 1999, 285, 1028.10446041

[26] M. J. Humphries , P. A. McEwan , S. J. Barton , P. A. Buckley , J. Bella , A. P. Mould , Trends Biochem. Sci. 2003, 28, 313. 12826403 10.1016/s0968-0004(03)00112-9

[27] H. Hamidi , J. Ivaska , Nat. Rev. Cancer 2018, 18, 533. 30002479 10.1038/s41568-018-0038-z PMC6629548

[28] D. Vestweber , Nat. Rev. Immunol. 2015, 15, 692. 26471775 10.1038/nri3908

[29] J. Z. Kechagia , J. Ivaska , P. Roca‐Cusachs , Nat. Rev. Mol. Cell Biol. 2019, 20, 457. 31182865 10.1038/s41580-019-0134-2

[30] M. L. Dustin , Cell 2019, 177, 499. 30952447 10.1016/j.cell.2019.03.038

[31] Y. Hu , H. Li , W. Wang , F. Sun , C. Wu , W. Chen , Z. Liu , Nano Lett. 2023, 23, 5562. 37289965 10.1021/acs.nanolett.3c00957

[32] A. Gerard , A. P. Cope , C. Kemper , R. Alon , R. Kochl , Trends Immunol. 2021, 42, 706. 34266767 10.1016/j.it.2021.06.004 PMC10734378

[33] M. S. Wang , Y. Hu , E. E. Sanchez , X. Xie , N. H. Roy , M. de Jesus , B. Y. Winer , E. A. Zale , W. Jin , C. Sachar , J. H. Lee , Y. Hong , M. Kim , L. C. Kam , K. Salaita , M. Huse , Nat. Commun. 2022, 13, 3222. 35680882 10.1038/s41467-022-30809-3 PMC9184626

[34] V. Ma , Y. Hu , A. Kellner , J. Brockman , A. Velusamy , A. Blanchard , B. Evavold , R. Alon , K. Salaita , Sci. Adv. 2022, 8, abg4485. 10.1126/sciadv.abg4485 PMC8880789 35213231

[35] Z. Huo , W. Yang , J. Harati , A. Nene , F. Borghi , C. Piazzoni , P. Milani , S. Guo , M. Galluzzi , D. Boraschi , ACS Appl. Mater. Interfaces 2024, 16, 27164. 38750662 10.1021/acsami.4c04330

[36] Z. Zeng , E. Chen , J. Xue , Autoimmun. Rev. 2025, 103813. 40194731 10.1016/j.autrev.2025.103813

[37] A. Lai , C. D. Cox , N. Chandra Sekar , P. Thurgood , A. Jaworowski , K. Peter , S. Baratchi , Biol. Rev. Camb. Philos. Soc. 2022, 97, 604. 34781417 10.1111/brv.12814

[38] B. Xiao , Nat. Rev. Mol. Cell Biol. 2024, 25, 886. 39251883 10.1038/s41580-024-00773-5

[39] A. Saraswathibhatla , D. Indana , O. Chaudhuri , Nat. Rev. Mol. Cell Biol. 2023, 24, 495. 36849594 10.1038/s41580-023-00583-1 PMC10656994

[40] Z. Mai , Y. Lin , P. Lin , X. Zhao , L. Cui , Cell Death Dis. 2024, 15, 307. 38693104 10.1038/s41419-024-06697-4 PMC11063215

[41] J. M. Kefauver , A. B. Ward , A. Patapoutian , Nature 2020, 587, 567. 33239794 10.1038/s41586-020-2933-1 PMC8477435

[42] T. Zhao , Y. Huang , J. Zhu , Y. Qin , H. Wu , J. Yu , Q. Zhai , S. Li , X. Qin , D. Wang , T. Li , Y. Liu , MedComm. 2025, 6, 70281. 10.1002/mco2.70281 PMC12271642 40686923

[43] Y. Jiang , H. Zhang , J. Wang , Y. Liu , T. Luo , H. Hua , J. Hematol. Oncol. 2022, 15, 34. 35331296 10.1186/s13045-022-01252-0 PMC8943941

[44] L. Niu , B. Cheng , G. Huang , K. Nan , S. Han , H. Ren , N. Liu , Y. Li , G. M. Genin , F. Xu , Cell Discov. 2022, 8, 84. 36068215 10.1038/s41421-022-00427-w PMC9448780

[45] H. Atcha , A. Jairaman , J. R. Holt , V. S. Meli , R. R. Nagalla , P. K. Veerasubramanian , K. T. Brumm , H. E. Lim , S. Othy , M. D. Cahalan , M. M. Pathak , W. F. Liu , Nat. Commun. 2021, 12, 3256. 34059671 10.1038/s41467-021-23482-5 PMC8167181

[46] Y. Wang , J. Wang , J. Zhang , Y. Wang , Y. Wang , H. Kang , G. Zhao , W. Bai , N. Miao , J. Wang , Sci. Adv. 2024, 10, adj3289. 10.1126/sciadv.adj3289 PMC11152137 38838160

[47] M. Chakraborty , K. Chu , A. Shrestha , X. S. Revelo , X. Zhang , M. J. Gold , S. Khan , M. Lee , C. Huang , M. Akbari , F. Barrow , Y. T. Chan , H. Lei , N. K. Kotoulas , J. Jovel , C. Pastrello , M. Kotlyar , C. Goh , E. Michelakis , X. Clemente‐Casares , P. S. Ohashi , E. G. Engleman , S. Winer , I. Jurisica , S. Tsai , D. A. Winer , Cell Rep. 2021, 34, 108609. 33440149 10.1016/j.celrep.2020.108609

[48] S. Baratchi , H. Danish , C. Chheang , Y. Zhou , A. Huang , A. Lai , M. Khanmohammadi , K. M. Quinn , K. Khoshmanesh , K. Peter , Nat. Commun. 2024, 15, 7023. 39174529 10.1038/s41467-024-51211-1 PMC11341855

[49] A. Fish , A. Kulkarni , ACS Appl. Mater. Interfaces 2024, 16, 4505. 38240257 10.1021/acsami.3c18645 PMC12965337

[50] A. Mukhopadhyay , Y. Tsukasaki , W. C. Chan , J. P. Le , M. L. Kwok , J. Zhou , V. Natarajan , N. Mostafazadeh , M. Maienschein‐Cline , I. Papautsky , C. Tiruppathi , Z. Peng , J. Rehman , B. Ganesh , Y. Komarova , A. B. Malik , Immunity 2024, 57, 52. 38091995 10.1016/j.immuni.2023.11.007 PMC10872880

[51] A. Jairaman , S. Othy , J. L. Dynes , A. Zavala , M. L. Greenberg , J. L. Nourse , J. R. Holt , S. M. Cahalan , F. Marangoni , L. Parker , M. M. Pathak , M. D. Cahalan , Sci. Adv. 2021, 7, abg5859. 10.1126/sciadv.abg5859 PMC8262815 34233878

[52] Q. Yang , Y. Cao , L. Wang , Y. Dong , L. Zhao , Z. Geng , Y. Bi , G. Liu , Cell Rep. 2025, 44, 115136. 39932192 10.1016/j.celrep.2024.115136

[53] Q. Wang , Y. Qin , B. Li , Cancer Lett. 2023, 559, 216043. 36584935 10.1016/j.canlet.2022.216043

[54] R. Pang , W. Sun , Y. Yang , D. Wen , F. Lin , D. Wang , K. Li , N. Zhang , J. Liang , C. Xiong , Y. Liu , Nat. Biomed. Eng. 2024, 8, 1162. 38514773 10.1038/s41551-024-01188-5

[55] P. K. Randhawa , A. S. Jaggi , Basic Res. Cardiol. 2015, 110, 54. 26415881 10.1007/s00395-015-0512-7

[56] K. Poole , Annu. Rev. Physiol. 2022, 84, 307. 34637325 10.1146/annurev-physiol-060721-100935

[57] H. Bai , L. Si , A. Jiang , C. Belgur , Y. Zhai , R. Plebani , C. Y. Oh , M. Rodas , A. Patil , A. Nurani , S. E. Gilpin , R. K. Powers , G. Goyal , R. Prantil‐Baun , D. E. Ingber , Nat. Commun. 2022, 13, 1928. 35396513 10.1038/s41467-022-29562-4 PMC8993817

[58] B. Dutta , R. Goswami , S. O. Rahaman , Front Immunol. 2020, 11, 570195. 33381111 10.3389/fimmu.2020.570195 PMC7767862

[59] N. R. Wong , J. Mohan , B. J. Kopecky , S. Guo , L. Du , J. Leid , G. Feng , I. Lokshina , O. Dmytrenko , H. Luehmann , G. Bajpai , L. Ewald , L. Bell , N. Patel , A. Bredemeyer , C. J. Weinheimer , J. M. Nigro , A. Kovacs , S. Morimoto , P. O. Bayguinov , M. R. Fisher , W. T. Stump , M. Greenberg , J. A. J. Fitzpatrick , S. Epelman , D. Kreisel , R. Sah , Y. Liu , H. Hu , K. J. Lavine , Immunity 2021, 54, 2072. 34320366 10.1016/j.immuni.2021.07.003 PMC8446343

[60] M. Dogterom , G. H. Koenderink , Nat. Rev. Mol. Cell Biol. 2019, 20, 38. 30323238 10.1038/s41580-018-0067-1

[61] D. Huang , S. Chen , D. Xiong , H. Wang , L. Zhu , Y. Wei , Y. Li , S. Zou , Aging Dis. 2023, 14, 1511. 37196113 10.14336/AD.2023.0201 PMC10529762

[62] S. Mylvaganam , S. A. Freeman , S. Grinstein , Curr. Biol. 2021, 31, R619. 34033794 10.1016/j.cub.2021.01.036

[63] E. G. G. Sprenkeler , A. T. J. Tool , S. S. V. Henriet , R. van Bruggen , T. W. Kuijpers , Blood 2022, 139, 3166. 35030250 10.1182/blood.2021013565

[64] J. S. Park , C. J. Burckhardt , R. Lazcano , L. M. Solis , T. Isogai , L. Li , C. S. Chen , B. Gao , J. D. Minna , R. Bachoo , R. J. DeBerardinis , G. Danuser , Nature 2020, 578, 621. 32051585 10.1038/s41586-020-1998-1 PMC7210009

[65] J. Geng , Y. Shi , J. Zhang , B. Yang , P. Wang , W. Yuan , H. Zhao , J. Li , F. Qin , L. Hong , C. Xie , X. Deng , Y. Sun , C. Wu , L. Chen , D. Zhou , Nat. Commun. 2021, 12, 3519. 34112781 10.1038/s41467-021-23683-y PMC8192512

[66] P. Tolar , Nat. Rev. Immunol. 2017, 17, 621. 28690317 10.1038/nri.2017.67

[67] A. Kumari , J. Pineau , P. J. Saez , M. Maurin , D. Lankar , M. San Roman , K. Hennig , V. F. Boura , R. Voituriez , M. C. I. Karlsson , M. Balland , A. M. Lennon Dumenil , P. Pierobon , Nat. Commun. 2019, 10, 2870. 31253773 10.1038/s41467-019-10751-7 PMC6599028

[68] J. C. Wang , Y. I. Yim , X. Wu , V. Jaumouille , A. Cameron , C. M. Waterman , J. H. Kehrl , J. A. Hammer , Elife 2022, 11, 72805. 10.7554/eLife.72805 PMC9142150 35404237

[69] M. Meizlish , Y. Kimura , S. Pope , R. Matta , C. Kim , N. Philip , L. Meyaard , A. Gonzalez , R. Medzhitov , Sci. Adv. 2024, 10, adk6906. 10.1126/sciadv.adk6906 PMC10936955 38478620

[70] S. Piccolo , S. Dupont , M. Cordenonsi , Physiol. Rev. 2014, 94, 1287. 25287865 10.1152/physrev.00005.2014

[71] Y. Wei , V. L. Z. Hui , Y. Chen , R. Han , X. Han , Y. Guo , MedComm. 2023, 4, 340. 10.1002/mco2.340 PMC10412783 37576865

[72] I. M. Moya , G. Halder , Nat. Rev. Mol. Cell Biol. 2019, 20, 211. 30546055 10.1038/s41580-018-0086-y

[73] F. Zanconato , M. Cordenonsi , S. Piccolo , Cancer Cell 2016, 29, 783. 27300434 10.1016/j.ccell.2016.05.005 PMC6186419

[74] M. Aragona , T. Panciera , A. Manfrin , S. Giulitti , F. Michielin , N. Elvassore , S. Dupont , S. Piccolo , Cell 2013, 154, 1047. 23954413 10.1016/j.cell.2013.07.042

[75] M. Ji , D. Chen , Y. Shu , S. Dong , Z. Zhang , H. Zheng , X. Jin , L. Zheng , Y. Liu , Y. Zheng , W. Zhang , S. Wang , G. Zhou , B. Li , B. Ji , Y. Yang , Y. Xu , L. Chang , Nat. Commun. 2023, 14, 3758. 37353497 10.1038/s41467-023-39009-z PMC10290143

[76] L. Liu , Y. Wang , S. Yu , H. Liu , Y. Li , S. Hua , Y. G. Chen , Adv. Sci. (Weinh) 2023, 10, 2300708. 37261975 10.1002/advs.202300708 PMC10427365

[77] Y. Wang , X. Geng , Z. Guo , D. Chu , R. Liu , B. Cheng , H. Cui , C. Li , J. Li , Z. Li , Ann. Med. 2024, 56, 2313680. 38335557 10.1080/07853890.2024.2313680 PMC10860428

[78] M. M. Mia , D. M. Cibi , S. A. B. Abdul Ghani , W. Song , N. Tee , S. Ghosh , J. Mao , E. N. Olson , M. K. Singh , PLoS Biol. 2020, 18, 3000941. 10.1371/journal.pbio.3000941 PMC7735680 33264286

[79] K. P. Meng , F. S. Majedi , T. J. Thauland , M. J. Butte , J. Exp. Med. 2020, 217, 20200053. 10.1084/jem.20200053 PMC7398163 32484502

[80] V. Ramjee , D. Li , L. J. Manderfield , F. Liu , K. A. Engleka , H. Aghajanian , C. B. Rodell , W. Lu , V. Ho , T. Wang , L. Li , A. Singh , D. M. Cibi , J. A. Burdick , M. K. Singh , R. Jain , J. A. Epstein , J. Clin. Invest. 2017, 127, 899. 28165342 10.1172/JCI88759 PMC5330722

[81] S. Dupont , L. Morsut , M. Aragona , E. Enzo , S. Giulitti , M. Cordenonsi , F. Zanconato , J. Le Digabel , M. Forcato , S. Bicciato , N. Elvassore , S. Piccolo , Nature 2011, 474, 179. 21654799 10.1038/nature10137

[82] S. P. Nobs , A. A. Kolodziejczyk , L. Adler , N. Horesh , C. Botscharnikow , E. Herzog , G. Mohapatra , S. Hejndorf , R. J. Hodgetts , I. Spivak , L. Schorr , L. Fluhr , D. Kviatcovsky , A. Zacharia , S. Njuki , D. Barasch , N. Stettner , M. Dori‐Bachash , A. Harmelin , A. Brandis , T. Mehlman , A. Erez , Y. He , S. Ferrini , J. Puschhof , H. Shapiro , M. Kopf , A. Moussaieff , S. K. Abdeen , E. Elinav , Nature 2023, 624, 645. 38093014 10.1038/s41586-023-06803-0 PMC10733144

[83] V. S. Meli , P. K. Veerasubramanian , T. L. Downing , W. Wang , W. F. Liu , Sci. Signaling 2023, 16, adc9656. 10.1126/scisignal.adc9656 PMC10625748 37130167

[84] J. C. Bahr , X. Y. Li , T. Y. Feinberg , L. Jiang , S. J. Weiss , Nat. Commun. 2022, 13, 6409. 36302921 10.1038/s41467-022-34087-x PMC9613642

[85] Z. Yuan , Y. Li , S. Zhang , X. Wang , H. Dou , X. Yu , Z. Zhang , S. Yang , M. Xiao , Mol. Cancer 2023, 22, 48. 36906534 10.1186/s12943-023-01744-8 PMC10007858

[86] R. Passariello , G. Imparato , C. Casale , F. Urciuolo , P. A. Netti , Acta Biomater. 2025, 204, 293. 40819725 10.1016/j.actbio.2025.08.027

[87] A. Dey , X. Varelas , K. L. Guan , Nat. Rev. Drug. Discov. 2020, 19, 480. 32555376 10.1038/s41573-020-0070-z PMC7880238

[88] A. Mantovani , C. Garlanda , N. Engl. J. Med. 2023, 388, 439. 36724330 10.1056/NEJMra2206346 PMC9912245

[89] A. G. Solis , P. Bielecki , H. R. Steach , L. Sharma , C. C. D. Harman , S. Yun , M. R. de Zoete , J. N. Warnock , S. D. F. To , A. G. York , M. Mack , M. A. Schwartz , C. S. Dela Cruz , N. W. Palm , R. Jackson , R. A. Flavell , Nature 2019, 573, 69. 31435009 10.1038/s41586-019-1485-8 PMC6939392

[90] S. Pourteymour , J. Fan , R. K. Majhi , S. Guo , X. Sun , Z. Huang , Y. Liu , H. Winter , A. Backlund , N. T. Skenteris , E. Chernogubova , O. Werngren , Z. Li , J. Skogsberg , Y. Li , L. Matic , U. Hedin , L. Maegdefessel , E. Ehrenborg , Y. Tian , H. Jin , Cell. Mol. Life Sci. 2024, 81, 331. 39107572 10.1007/s00018-024-05372-3 PMC11335255

[91] M. Locati , G. Curtale , A. Mantovani , Annu. Rev. Pathol. 2020, 15, 123. 31530089 10.1146/annurev-pathmechdis-012418-012718 PMC7176483

[92] Y. Xu , L. Ying , J. Lang , B. Hinz , R. Zhao , Sci. Adv. 2024, 10, adj9559. 10.1126/sciadv.adj9559 PMC10980276 38552026

[93] H. Yu , L. Lin , Z. Zhang , H. Zhang , H. Hu , Signal Transduct. Target Ther. 2020, 5, 209. 32958760 10.1038/s41392-020-00312-6 PMC7506548

[94] Z. Yang , Y. Zhao , X. Zhang , L. Huang , K. Wang , J. Sun , N. Chen , W. Yin , S. Chen , H. Zhi , L. Xue , L. An , R. Li , H. Dong , J. Xu , Y. Li , Y. Li , ACS Nano 2024, 18, 21221. 39079080 10.1021/acsnano.4c04614

[95] G. L. Burn , A. Foti , G. Marsman , D. F. Patel , A. Zychlinsky , The Neutrophil, Immunity 2021, 54, 1377. 34260886 10.1016/j.immuni.2021.06.006

[96] W. Gao , X. Zhang , W. Hu , J. Han , X. Liu , Y. Zhang , M. Long , Biomaterials 2025, 314, 122881. 39454506 10.1016/j.biomaterials.2024.122881

[97] T. Jiang , X. Y. Tang , Y. Mao , Y. Q. Zhou , J. J. Wang , R. M. Li , X. R. Xie , H. M. Zhang , B. Fang , N. J. Ouyang , G. H. Tang , Acta Biomater. 2023, 168, 159. 37467837 10.1016/j.actbio.2023.07.012

[98] J. O. Abaricia , A. H. Shah , R. Olivares‐Navarrete , Biomaterials 2021, 271, 120715. 33677375 10.1016/j.biomaterials.2021.120715 PMC8044006

[99] J. Idoyaga , R. M. Steinman , Cell 2011, 146, 660. 21854989 10.1016/j.cell.2011.08.010

[100] Y. Choi , J. E. Kwon , Y. K. Cho , Cells 2021, 10, 3362.34943870

[101] H. Yu , Z. Liu , H. Guo , X. Hu , Y. Wang , X. Cheng , L. W. Zhang , Y. Wang , ACS Nano 2024, 18, 23741. 39158207 10.1021/acsnano.4c08701

[102] B. Hu , Y. Xin , G. Hu , K. Li , Y. Tan , APL Bioeng. 2023, 7, 036108. 37575881 10.1063/5.0156628 PMC10423075

[103] J. Wu , D. Wu , G. Wu , H. P. Bei , Z. Li , H. Xu , Y. Wang , D. Wu , H. Liu , S. Shi , C. Zhao , Y. Xu , Y. He , J. Li , C. Wang , X. Zhao , S. Wang , Biofabrication 2022, 14, 045004. 10.1088/1758-5090/ac7eeb 35793612

[104] G. Santoni , C. Amantini , M. Santoni , F. Maggi , M. B. Morelli , A. Santoni , Front. Immunol. 2021, 12, 688918. 34335592 10.3389/fimmu.2021.688918 PMC8320435

[105] D. Friedman , P. Simmonds , A. Hale , L. Bere , N. W. Hodson , M. R. H. White , D. M. Davis , J. Cell Sci. 2021, 134. 10.1242/jcs.258570 PMC8077183 33712452

[106] G. R. N. , N. Engl. J. Med. 2019, 381, 1083. 31502768 10.1056/NEJMcibr1909387

[107] D. J. Fowell , M. Kim , Nat. Rev. Immunol. 2021, 21, 582. 33627851 10.1038/s41577-021-00507-0 PMC9380693

[108] X. Zhang , T. H. Kim , T. J. Thauland , H. Li , F. S. Majedi , C. Ly , Z. Gu , M. J. Butte , A. C. Rowat , S. Li , Curr. Opin. Biotechnol. 2020, 66, 236. 33007634 10.1016/j.copbio.2020.09.004 PMC7524653

[109] M. de Jesus , A. H. Settle , D. Vorselen , T. K. Gaetjens , M. Galiano , Y. Romin , E. Lee , Y. Y. Wong , T. M. Fu , E. Santosa , B. Y. Winer , F. Tamzalit , M. S. Wang , A. Santella , Z. Bao , J. C. Sun , P. Shah , J. A. Theriot , S. M. Abel , M. Huse , Sci. Immunol. 2024, 9, adj2898. 10.1126/sciimmunol.adj2898 PMC11826491 38941478

[110] T. J. Thauland , K. H. Hu , M. A. Bruce , M. J. Butte , Sci. Signal. 2017, 10, aah3737. 10.1126/scisignal.aah3737 PMC5854469 28270556

[111] K. Adu‐Berchie , Y. Liu , D. K. Y. Zhang , B. R. Freedman , J. M. Brockman , K. H. Vining , B. A. Nerger , A. Garmilla , D. J. Mooney , Nat. Biomed. Eng. 2023, 7, 1374. 37365267 10.1038/s41551-023-01052-y PMC10749992

[112] J. Zhang , J. Li , Y. Hou , Y. Lin , H. Zhao , Y. Shi , K. Chen , C. Nian , J. Tang , L. Pan , Y. Xing , H. Gao , B. Yang , Z. Song , Y. Cheng , Y. Liu , M. Sun , Y. Linghu , J. Li , H. Huang , Z. Lai , Z. Zhou , Z. Li , X. Sun , Q. Chen , D. Su , W. Li , Z. Peng , P. Liu , W. Chen , et al., Cell 2024, 187, 3409. 38744281 10.1016/j.cell.2024.04.023

[113] W. Zou , Nat. Rev. Immunol. 2006, 6, 295. 16557261 10.1038/nri1806

[114] L. Shi , J. Y. Lim , L. C. Kam , Biomaterials 2023, 292, 121928. 36455488 10.1016/j.biomaterials.2022.121928 PMC9772289

[115] K. M. Spillane , P. Tolar , J. Cell Biol. 2017, 216, 217. 27923880 10.1083/jcb.201607064 PMC5223605

[116] Z. Wan , C. Xu , X. Chen , H. Xie , Z. Li , J. Wang , X. Ji , H. Chen , Q. Ji , S. Shaheen , Y. Xu , F. Wang , Z. Tang , J. S. Zheng , W. Chen , J. Lou , W. Liu , J. Cell Biol. 2018, 217, 2565. 29685902 10.1083/jcb.201711055 PMC6028545

[117] A. Naba , Nat. Rev. Mol. Cell Biol. 2024, 25, 865. 39223427 10.1038/s41580-024-00767-3 PMC11931590

[118] C. Bonnans , J. Chou , Z. Werb , Nat. Rev. Mol. Cell Biol. 2014, 15, 786. 25415508 10.1038/nrm3904 PMC4316204

[119] J. D. Humphrey , E. R. Dufresne , M. A. Schwartz , Nat. Rev. Mol. Cell Biol. 2014, 15, 802. 25355505 10.1038/nrm3896 PMC4513363

[120] P. Kanchanawong , Nat. Rev. Mol. Cell Biol. 2023, 24, 142. 36168065 10.1038/s41580-022-00531-5 PMC9892292

[121] R. Zheng , K. Shen , S. Liang , Y. Lyu , S. Zhang , H. Dong , Y. Li , Y. Han , X. Zhao , Y. Zhang , P. Wang , R. Meng , S. Bai , J. Yang , G. Lu , J. Li , A. Yang , R. Zhang , B. Yan , Cell Mol. Immunol. 2024, 21, 1491. 39472748 10.1038/s41423-024-01228-9 PMC11606952

[122] A. H. Settle , B. Y. Winer , M. M. de Jesus , L. Seeman , Z. Wang , E. Chan , Y. Romin , Z. Li , M. M. Miele , R. C. Hendrickson , D. Vorselen , J. S. A. Perry , Nat. Commun. 2024, 15, 8182. 39294148 10.1038/s41467-024-52453-9 PMC11411054

[123] V. S. Meli , H. Atcha , P. K. Veerasubramanian , R. R. Nagalla , T. U. Luu , E. Y. Chen , C. F. Guerrero‐Juarez , K. Yamaga , W. Pandori , J. Y. Hsieh , T. L. Downing , D. A. Fruman , M. B. Lodoen , M. V. Plikus , W. Wang , W. F. Liu , Sci. Adv. 2020, 6, abb8471. 10.1126/sciadv.abb8471 PMC7717914 33277245

[124] Q. Lai , B. Li , L. Chen , Y. Zhou , H. Bao , H. Li , Acta Biomater. 2025, 192, 77. 39662715 10.1016/j.actbio.2024.12.021

[125] J. Sapudom , A. Alatoom , P. S. Tipay , J. C. Teo , Biomaterials 2025, 315, 122900. 39461060 10.1016/j.biomaterials.2024.122900

[126] E. H. Puttock , E. J. Tyler , M. Manni , E. Maniati , C. Butterworth , M. Burger Ramos , E. Peerani , P. Hirani , V. Gauthier , Y. Liu , G. Maniscalco , V. Rajeeve , P. Cutillas , C. Trevisan , M. Pozzobon , M. Lockley , J. Rastrick , H. Laubli , A. White , O. M. T. Pearce , Nat. Commun. 2023, 14, 2514. 37188691 10.1038/s41467-023-38093-5 PMC10185550

[127] C. Robertson , A. Sebastian , A. Hinckley , N. D. Rios‐Arce , W. F. Hynes , S. A. Edwards , W. He , N. R. Hum , E. K. Wheeler , G. G. Loots , M. A. Coleman , M. L. Moya , Biomaterials 2022, 282, 121378. 35121359 10.1016/j.biomaterials.2022.121378

[128] M. Vyas , S. Demehri , Trends Immunol. 2022, 43, 423. 35527181 10.1016/j.it.2022.04.004

[129] B. Voisin , V. Nadella , T. Doebel , S. Goel , K. Sakamoto , O. Ayush , J. H. Jo , M. C. Kelly , T. Kobayashi , J. X. Jiang , Y. Hu , C. Yan , K. Nagao , Immunity 2023, 56, 1561. 37402364 10.1016/j.immuni.2023.06.006 PMC10467568

[130] C. E. Witherel , K. Sao , B. K. Brisson , B. Han , S. W. Volk , R. J. Petrie , L. Han , K. L. Spiller , Biomaterials 2021, 269, 120667. 33450585 10.1016/j.biomaterials.2021.120667 PMC7870567

[131] A. Fischer , J. Wannemacher , S. Christ , T. Koopmans , S. Kadri , J. Zhao , M. Gouda , H. Ye , M. Muck‐Hausl , P. W. Krenn , H. G. Machens , R. Fassler , P. A. Neumann , S. M. Hauck , Y. Rinkevich , Nat. Immunol. 2022, 23, 518. 35354953 10.1038/s41590-022-01166-6 PMC8986538

[132] J. Haj‐Shomaly , A. Vorontsova , T. Barenholz‐Cohen , O. Levi‐Galibov , M. Devarasetty , M. Timaner , Z. Raviv , T. J. Cooper , S. Soker , P. Hasson , D. Weihs , R. Scherz‐Shouval , Y. Shaked , Cancer Res. 2022, 82, 278. 34666995 10.1158/0008-5472.CAN-21-1012 PMC7612244

[133] H. De Belly , E. K. Paluch , K. J. Chalut , Nat. Rev. Mol. Cell Biol. 2022, 23, 465. 35365816 10.1038/s41580-022-00472-z

[134] C. S. C. Liu , T. Mandal , P. Biswas , M. A. Hoque , P. Bandopadhyay , B. P. Sinha , J. Sarif , R. D'Rozario , D. K. Sinha , B. Sinha , D. Ganguly , eLife 2024, 12, RP91903. 38393325 10.7554/eLife.91903 PMC10942591

[135] H. Atcha , V. S. Meli , C. T. Davis , K. T. Brumm , S. Anis , J. Chin , K. Jiang , M. M. Pathak , W. F. Liu , Front. Immunol. 2021, 12, 689397. 34630381 10.3389/fimmu.2021.689397 PMC8493066

[136] Y. Wang , Q. Yang , Y. Dong , L. Wang , Z. Zhang , R. Niu , Y. Wang , Y. Bi , G. Liu , Cell Death Dis. 2025, 16, 60. 39890818 10.1038/s41419-025-07395-5 PMC11785962

[137] Y. Wang , H. Yang , A. Jia , Y. Wang , Q. Yang , Y. Dong , Y. Hou , Y. Cao , L. Dong , Y. Bi , G. Liu , eLife 2022, 11, 79957. 10.7554/eLife.79957 PMC9451538 35993548

[138] N. K. Livingston , J. W. Hickey , H. Sim , S. F. Salathe , J. Choy , J. Kong , A. B. Silver , J. L. Stelzel , M. O. Omotoso , S. Li , W. Chaisawangwong , S. Roy , E. C. Ariail , M. R. Lanis , P. Pradeep , J. G. Bieler , S. E. Witte , E. Leonard , J. C. Doloff , J. B. Spangler , H. Q. Mao , J. P. Schneck , Adv. Mater. 2024, 36, 2310043. 10.1002/adma.202310043 PMC11161322 38358310

[139] J. Lou , C. Meyer , E. B. Vitner , K. Adu‐Berchie , M. T. Dacus , G. Bovone , A. Chen , T. To , D. A. Weitz , D. J. Mooney , Adv. Mater. 2024, 36, 2309860. 10.1002/adma.202309860 PMC11293993 38615189

[140] Y. Du , Y. Lyu , J. Lin , C. Ma , Q. Zhang , Y. Zhang , L. Qiu , W. Tan , Nat. Nanotechnol. 2023, 18, 818. 36894782 10.1038/s41565-023-01333-2

[141] M. L. Dustin , J. Clin. Invest. 2012, 122, 1149. 22466656 10.1172/JCI58705 PMC3314453

[142] S. Kumari , M. Mak , Y. C. Poh , M. Tohme , N. Watson , M. Melo , E. Janssen , M. Dustin , R. Geha , D. J. Irvine , EMBO J. 2020, 39, 102783. 10.15252/embj.2019102783 PMC7049817 31894880

[143] S. Camargo , O. Moskowitz , A. Giladi , M. Levinson , R. Balaban , S. Gola , A. Raizman , K. Lipczyc , A. Richter , N. Keren‐Khadmy , O. Barboy , Y. Dugach , Y. Carmi , A. Sonnenblick , M. Cohen , Nat. Cancer 2025, 6, 540. 40055573 10.1038/s43018-025-00924-3

[144] R. Qin , Y. Zhang , J. Shi , P. Wu , C. An , Z. Li , N. Liu , Z. Wan , T. Hua , X. Li , J. Lou , W. Yin , W. Chen , Cell Res. 2025, 35, 265. 40011760 10.1038/s41422-025-01077-9 PMC11958657

[145] Y. Liu , T. Zhang , H. Zhang , J. Li , N. Zhou , R. Fiskesund , J. Chen , J. Lv , J. Ma , H. Zhang , K. Tang , F. Cheng , Y. Zhou , X. Zhang , N. Wang , B. Huang , Cancer Res. 2021, 81, 476. 33168645 10.1158/0008-5472.CAN-20-2569

[146] X. Zhou , R. A. Franklin , M. Adler , T. S. Carter , E. Condiff , T. S. Adams , S. D. Pope , N. H. Philip , M. L. Meizlish , N. Kaminski , R. Medzhitov , Proc. Natl. Acad Sci. USA 2022, 119, 2205360119. 10.1073/pnas.2205360119 PMC9371703 35930670

[147] J. S. Choi , B. A. Harley , Sci. Adv. 2017, 3, 1600455. 10.1126/sciadv.1600455 PMC5218514 28070554

[148] G. Shi , Z. Chang , P. Zhang , X. Zou , X. Zheng , X. Liu , J. Yan , H. Xu , Z. Tian , N. Zhang , N. Cui , L. Sun , G. Xu , H. Yang , Cell Prolif 2024, 57, 13715. 10.1111/cpr.13715 PMC11628730 38982593

[149] E. M. Kernfeld , R. M. J. Genga , K. Neherin , M. E. Magaletta , P. Xu , R. Maehr , Immunity 2018, 48, 1258e1256. 29884461 10.1016/j.immuni.2018.04.015 PMC6013397

[150] M. A. Asnaghi , T. Barthlott , F. Gullotta , V. Strusi , A. Amovilli , K. Hafen , G. Srivastava , P. Oertle , R. Toni , D. Wendt , G. A. Hollander , I. Martin , Adv. Funct. Mater. 2021, 31, 2010747. 34539304 10.1002/adfm.202010747 PMC8436951

[151] J. Hong , C. Ge , P. Jothikumar , Z. Yuan , B. Liu , K. Bai , K. Li , W. Rittase , M. Shinzawa , Y. Zhang , A. Palin , P. Love , X. Yu , K. Salaita , B. D. Evavold , A. Singer , C. Zhu , Nat. Immunol. 2018, 19, 1379. 30420628 10.1038/s41590-018-0259-z PMC6452639

[152] Y. Kamioka , Y. Ueda , N. Kondo , K. Tokuhiro , Y. Ikeda , W. Bergmeier , T. Kinashi , Cell Rep. 2023, 42, 112580. 37267105 10.1016/j.celrep.2023.112580 PMC10592472

[153] Z. Shulman , V. Shinder , E. Klein , V. Grabovsky , O. Yeger , E. Geron , A. Montresor , M. Bolomini‐Vittori , S. W. Feigelson , T. Kirchhausen , C. Laudanna , G. Shakhar , R. Alon , Immunity 2009, 30, 384. 19268609 10.1016/j.immuni.2008.12.020 PMC2803105

[154] G. A. Dominguez , N. R. Anderson , D. A. Hammer , Integr. Biol. 2015, 7, 345. 10.1039/c4ib00201f PMC4746477 25674729

[155] D. Furman , J. Campisi , E. Verdin , P. Carrera‐Bastos , S. Targ , C. Franceschi , L. Ferrucci , D. W. Gilroy , A. Fasano , G. W. Miller , A. H. Miller , A. Mantovani , C. M. Weyand , N. Barzilai , J. J. Goronzy , T. A. Rando , R. B. Effros , A. Lucia , N. Kleinstreuer , G. M. Slavich , Nat. Med. 2019, 25, 1822. 31806905 10.1038/s41591-019-0675-0 PMC7147972

[156] J. P. Campbell , N. E. Riddell , V. E. Burns , M. Turner , J. J. van Zanten , M. T. Drayson , J. A. Bosch , Brain Behav. Immun. 2009, 23, 767. 19254756 10.1016/j.bbi.2009.02.011

[157] J. Rehman , P. J. Mills , S. M. Carter , J. Chou , J. Thomas , A. S. Maisel , Brain, Behav., Immun. 1997, 11, 343. 9512820 10.1006/brbi.1997.0498

[158] J. A. B. Smith , K. A. Murach , K. A. Dyar , J. R. Zierath , Nat. Rev. Mol. Cell Biol. 2023, 24, 607. 37225892 10.1038/s41580-023-00606-x PMC10527431

[159] M. Flockhart , L. C. Nilsson , S. Tais , B. Ekblom , W. Apro , F. J. Larsen , Cell Metab. 2021, 33, 957. 33740420 10.1016/j.cmet.2021.02.017

[160] P. K. Langston , Y. Sun , B. A. Ryback , A. L. Mueller , B. M. Spiegelman , C. Benoist , D. Mathis , Sci. Immunol. 2023, 8, adi5377. 10.1126/sciimmunol.adi5377 PMC10860652 37922340

[161] S. Wang , H. Yan , B. Fang , C. Gu , J. Guo , P. Qiu , N. Song , W. Xu , J. Zhang , X. Lin , X. Fang , Biomaterials 2022, 285, 121519. 35552116 10.1016/j.biomaterials.2022.121519

[162] M. L. Gertz , C. R. Chin , D. Tomoiaga , M. MacKay , C. Chang , D. Butler , E. Afshinnekoo , D. Bezdan , M. A. Schmidt , C. Mozsary , A. Melnick , F. Garrett‐Bakelman , B. Crucian , S. M. C. Lee , S. R. Zwart , S. M. Smith , C. Meydan , C. E. Mason , Cell Rep. 2020, 33, 108429. 33242408 10.1016/j.celrep.2020.108429 PMC9444344

[163] E. Mitsou , J. Klein , Small 2025, 21, 2410060. 40143645 10.1002/smll.202410060 PMC12036560

[164] E. Sanchez‐Lopez , R. Coras , A. Torres , N. E. Lane , M. Guma , Nat. Rev. Rheumatol. 2022, 18, 258. 35165404 10.1038/s41584-022-00749-9 PMC9050956

[165] O. S. Svendsen , M. M. Barczyk , S. N. Popova , A. Liden , D. Gullberg , H. Wiig , Arterioscler Thromb. Vasc. Biol. 2009, 29, 1864. 19729609 10.1161/ATVBAHA.109.194308

[166] P. H. Zhang , W. W. Zhang , S. S. Wang , C. H. Wu , Y. D. Ding , X. Y. Wu , F. G. Smith , Y. Hao , S. W. Jin , JCI Insight 2024, 9. 10.1172/jci.insight.173440 PMC10906459 37971881

[167] L. S. B. Boisserand , L. H. Geraldo , J. Bouchart , M. R. El Kamouh , S. Lee , B. G. Sanganahalli , M. Spajer , S. Zhang , S. Lee , M. Parent , Y. Xue , M. Skarica , X. Yin , J. Guegan , K. Boye , F. Saceanu Leser , L. Jacob , M. Poulet , M. Li , X. Liu , S. E. Velazquez , R. Singhabahu , M. E. Robinson , M. H. Askenase , A. Osherov , N. Sestan , J. Zhou , K. Alitalo , E. Song , A. Eichmann , et al., J. Exp. Med. 2024, 221, 20221983. 10.1084/jem.20221983 PMC10913814 38442272

[168] A. Hunziker , I. Glas , M. O. Pohl , S. Stertz , Cell Rep. 2022, 38, 110306. 35081340 10.1016/j.celrep.2022.110306

[169] L. Du , Y. N. Hou , D. D. Fu , J. Li , J. Ao , A. X. Ma , Q. Q. Wan , Z. G. Wang , S. L. Liu , L. J. Zhang , D. W. Pang , ACS Nano 2024, 18, 23090. 39143650 10.1021/acsnano.4c05261

[170] A. Cont , T. Rossy , Z. Al‐Mayyah , A. Persat , Elife 2020, 9. 10.7554/eLife.56533 PMC7556879 33025904

[171] L. Wang , Y. C. Wong , J. M. Correira , M. Wancura , C. J. Geiger , S. S. Webster , A. Touhami , B. J. Butler , G. A. O'Toole , R. M. Langford , K. A. Brown , B. Dortdivanlioglu , L. Webb , E. Cosgriff‐Hernandez , V. D. Gordon , NPJ Biofilms Microbiomes 2023, 9, 78. 37816780 10.1038/s41522-023-00436-x PMC10564899

[172] J. A. Kochanowski , B. Carroll , M. E. Asp , E. C. Kaputa , A. E. Patteson , ACS Appl. Bio Mater. 2024, 7, 7809. 10.1021/acsabm.3c00907 PMC11653398 38193703

[173] J. Nijjer , C. Li , Q. Zhang , H. Lu , S. Zhang , J. Yan , Nat. Commun. 2021, 12, 6632. 34789754 10.1038/s41467-021-26869-6 PMC8599862

[174] I. Heras‐Murillo , I. Adan‐Barrientos , M. Galan , S. K. Wculek , D. Sancho , Nat. Rev. Clin. Oncol. 2024, 21, 257. 38326563 10.1038/s41571-024-00859-1

[175] G. Oliveira , C. J. Wu , Nat. Rev. Cancer 2023, 23, 295. 37046001 10.1038/s41568-023-00560-y PMC10773171

[176] T. J. Laskowski , A. Biederstadt , K. Rezvani , Nat. Rev. Cancer 2022, 22, 557. 35879429 10.1038/s41568-022-00491-0 PMC9309992

[177] L. Liu , X. Cheng , H. Yang , S. Lian , Y. Jiang , J. Liang , X. Chen , S. Mo , Y. Shi , S. Zhao , J. Li , R. Jiang , D. H. Yang , Y. Wu , Mol. Cancer 2022, 21, 59. 35193595 10.1186/s12943-022-01516-w PMC8862474

[178] R. Rui , L. Zhou , S. He , Front. Immunol. 2023, 14, 1212476. 37691932 10.3389/fimmu.2023.1212476 PMC10484345

[179] X. Liu , N. Ye , S. Liu , J. Guan , Q. Deng , Z. Zhang , C. Xiao , Z. Y. Ding , B. X. Zhang , X. P. Chen , Z. Li , X. Yang , Adv. Sci. 2021, 8, 2100233. 10.1002/advs.202100233 PMC8336507 34085419

[180] K. Pang , Z. D. Shi , L. Y. Wei , Y. Dong , Y. Y. Ma , W. Wang , G. Y. Wang , M. Y. Cao , J. J. Dong , Y. A. Chen , P. Zhang , L. Hao , H. Xu , D. Pan , Z. S. Chen , C. H. Han , Drug Resist. Updat. 2023, 66, 100907. 36527888 10.1016/j.drup.2022.100907

[181] A. Ribas , J. D. Wolchok , Science 2018, 359, 1350. 29567705 10.1126/science.aar4060 PMC7391259

[182] S. Schlichtner , I. M. Yasinska , G. S. Lall , S. M. Berger , S. Ruggiero , D. Cholewa , N. Aliu , B. F. Gibbs , E. Fasler‐Kan , V. V. Sumbayev , J. Immunother Cancer 2023, 11. 10.1136/jitc-2022-005714 PMC9815087 36599470

[183] C. Wang , X. Zheng , J. Zhang , X. Jiang , J. Wang , Y. Li , X. Li , G. Shen , J. Peng , P. Zheng , Y. Gu , J. Chen , M. Lin , C. Deng , H. Gao , Z. Lu , Y. Zhao , M. Luo , Nature 2023, 621, 830. 37674079 10.1038/s41586-023-06511-9

[184] J. Hyun , H. W. Kim , Trends Mol. Med. 2022, 28, 155. 34973934 10.1016/j.molmed.2021.11.006

[185] J. Cooper , F. G. Giancotti , Cancer Cell 2019, 35, 347. 30889378 10.1016/j.ccell.2019.01.007 PMC6684107

[186] B. Aykut , R. Chen , J. I. Kim , D. Wu , S. A. A. Shadaloey , R. Abengozar , P. Preiss , A. Saxena , S. Pushalkar , J. Leinwand , B. Diskin , W. Wang , G. Werba , M. Berman , S. K. B. Lee , A. Khodadadi‐Jamayran , D. Saxena , W. A. Coetzee , G. Miller , Science immunology 2020, 5, abb5168. 10.1126/sciimmunol.abb5168 32826342

[187] F. Zanconato , M. Cordenonsi , S. Piccolo , Nat. Rev. Cancer 2019, 19, 454. 31270418 10.1038/s41568-019-0168-y

[188] X. Ni , J. Tao , J. Barbi , Q. Chen , B. V. Park , Z. Li , N. Zhang , A. Lebid , A. Ramaswamy , P. Wei , Y. Zheng , X. Zhang , X. Wu , P. Vignali , C. P. Yang , H. Li , D. Pardoll , L. Lu , D. Pan , F. Pan , Cancer Discov. 2018, 8, 1026. 29907586 10.1158/2159-8290.CD-17-1124 PMC6481611

[189] Y. Zhou , D. Wang , L. Zhou , N. Zhou , Z. Wang , J. Chen , R. Pang , H. Fu , Q. Huang , F. Dong , H. Cheng , H. Zhang , K. Tang , J. Ma , J. Lv , T. Cheng , R. Fiskesund , X. Zhang , B. Huang , Nat. Commun. 2024, 15, 1405. 38360940 10.1038/s41467-024-45750-w PMC10869718

[190] J. Hyun , S. J. Kim , S. D. Cho , H. W. Kim , Biomaterials 2023, 297, 122101. 37023528 10.1016/j.biomaterials.2023.122101

[191] D. Lv , Y. Fei , H. Chen , J. Wang , W. Han , B. Cui , Y. Feng , P. Zhang , J. Chen , Front. Immunol. 2024, 15, 1340702. 38690275 10.3389/fimmu.2024.1340702 PMC11058664

[192] D. E. Kuczek , A. M. H. Larsen , M. L. Thorseth , M. Carretta , A. Kalvisa , M. S. Siersbaek , A. M. C. Simoes , A. Roslind , L. H. Engelholm , E. Noessner , M. Donia , I. M. Svane , P. T. Straten , L. Grontved , D. H. Madsen , J. Immunother Cancer 2019, 7, 68. 30867051 10.1186/s40425-019-0556-6 PMC6417085

[193] A. M. Fuller , H. C. Pruitt , Y. Liu , V. M. Irizarry‐Negron , H. Pan , H. Song , A. DeVine , R. S. Katti , S. Devalaraja , G. E. Ciotti , M. V. Gonzalez , E. F. Williams , I. Murazzi , D. Ntekoumes , N. Skuli , H. Hakonarson , D. J. Zabransky , J. G. Trevino , A. Weeraratna , K. Weber , M. Haldar , J. A. Fraietta , S. Gerecht , T. S. K. Eisinger‐Mathason , J. Clin. Invest. 2024, 134, 11. 10.1172/JCI167826 PMC11142734 38652549

[194] M. Lee , H. Du , D. A. Winer , X. Clemente‐Casares , S. Tsai , Front. Cell Dev. Biol. 2022, 10, 1044729. 36467420 10.3389/fcell.2022.1044729 PMC9712790

[195] M. Ali , Z. Foldvari , E. Giannakopoulou , M. L. Boschen , E. Stronen , W. Yang , M. Toebes , B. Schubert , O. Kohlbacher , T. N. Schumacher , J. Olweus , Nat. Protoc. 2019, 14, 1926. 31101906 10.1038/s41596-019-0170-6

[196] M. Peng , Y. Mo , Y. Wang , P. Wu , Y. Zhang , F. Xiong , C. Guo , X. Wu , Y. Li , X. Li , G. Li , W. Xiong , Z. Zeng , Mol Cancer 2019, 18, 128. 31443694 10.1186/s12943-019-1055-6 PMC6708248

[197] M. Bhattacharya , P. Ramachandran , Nat. Immunol. 2023, 24, 1423. 37474654 10.1038/s41590-023-01551-9

[198] Z. Xiao , E. Pure , Nat. Rev. Cancer 2025, 399. 40097577 10.1038/s41568-025-00798-8

[199] S. E. Mutsaers , T. Miles , C. M. Prele , G. F. Hoyne , Pharmacol. Ther. 2023, 252, 108562. 37952904 10.1016/j.pharmthera.2023.108562

[200] N. C. Henderson , F. Rieder , T. A. Wynn , Nature 2020, 587, 555. 33239795 10.1038/s41586-020-2938-9 PMC8034822

[201] C. Cohen , R. Mhaidly , H. Croizer , Y. Kieffer , R. Leclere , A. Vincent‐Salomon , C. Robley , D. Anglicheau , M. Rabant , A. Sannier , M. O. Timsit , S. Eddy , M. Kretzler , W. Ju , F. Mechta‐Grigoriou , Nat. Commun. 2024, 15, 743. 38272907 10.1038/s41467-024-44886-z PMC10810789

[202] P. F. Ma , C. C. Gao , J. Yi , J. L. Zhao , S. Q. Liang , Y. Zhao , Y. C. Ye , J. Bai , Q. J. Zheng , K. F. Dou , H. Han , H. Y. Qin , J. Hepatol 2017, 67, 770. 28596109 10.1016/j.jhep.2017.05.022

[203] M. Mabire , P. Hegde , A. Hammoutene , J. Wan , C. Caer , R. A. Sayegh , M. Cadoux , M. Allaire , E. Weiss , T. Thibault‐Sogorb , O. Lantz , M. Goodhardt , V. Paradis , P. de la Grange , H. Gilgenkrantz , S. Lotersztajn , Nat. Commun. 2023, 14, 1830. 37005415 10.1038/s41467-023-37453-5 PMC10067815

[204] Q. Ouyang , C. Wang , T. Sang , Y. Tong , J. Zhang , Y. Chen , X. Wang , L. Wu , X. Wang , R. Liu , P. Chen , J. Liu , W. Shen , Z. Feng , L. Zhang , X. Sun , G. Cai , L. L. Li , X. Chen , Cell Mol. Immunol. 2024, 21, 826. 38871810 10.1038/s41423-024-01190-6 PMC11291639

[205] L. E. Crowley , R. A. Stockley , D. R. Thickett , D. Dosanjh , A. Scott , D. Parekh , Eur. Respir. Rev. 2024, 33, 174. 10.1183/16000617.0139-2024 PMC11600124 39603661

[206] L. Luo , S. Wang , Y. Hu , L. Wang , X. Jiang , J. Zhang , X. Liu , X. Guo , Z. Luo , C. Zhu , M. Xie , Y. Li , J. You , F. Yang , ACS Nano 2023, 17, 22508. 37948096 10.1021/acsnano.3c05998

[207] E. Batlle , J. Massague , Immunity 2019, 50, 924. 30995507 10.1016/j.immuni.2019.03.024 PMC7507121

[208] L. Z. Rao , Y. Wang , L. Zhang , G. Wu , L. Zhang , F. X. Wang , L. M. Chen , F. Sun , S. Jia , S. Zhang , Q. Yu , J. H. Wei , H. R. Lei , T. Yuan , J. Li , X. Huang , B. Cheng , J. Zhao , Y. Xu , B. W. Mo , C. Y. Wang , H. Zhang , Cell Death Differ. 2021, 28, 1270. 33144678 10.1038/s41418-020-00650-6 PMC8027679

[209] S. Luo , X. Zhao , J. Jiang , B. Deng , S. Liu , H. Xu , Q. Tan , Y. Chen , Z. Zhang , X. Pan , R. Wan , X. Chen , Y. Yao , J. Li , Theranostics 2023, 13, 5418. 37908726 10.7150/thno.86103 PMC10614683

